# Investigating gut microbiota–blood and urine metabolite correlations in early sepsis-induced acute kidney injury: insights from targeted KEGG analyses

**DOI:** 10.3389/fcimb.2024.1375874

**Published:** 2024-06-03

**Authors:** Yaya Xu, Jiayue Xu, Yueniu Zhu, Haoyun Mao, Jiru Li, Xiangmei Kong, Xiaodong Zhu, Jianhua Zhang

**Affiliations:** ^1^ Department of Pediatric Critical Care Medicine, Xinhua Hospital, Affiliated to the Medical School of Shanghai Jiaotong University, Shanghai, China; ^2^ Department of Pediatric Respiratory, Xinhua Hospital, Affiliated to the Medical School of Shanghai Jiaotong University, Shanghai, China

**Keywords:** pediatric, kidney injury, sepsis, gut microbiota, blood metabolome

## Abstract

**Background:**

The interplay between gut microbiota and metabolites in the early stages of sepsis-induced acute kidney injury (SA-AKI) is not yet clearly understood. This study explores the characteristics and interactions of gut microbiota, and blood and urinary metabolites in patients with SA-AKI.

**Methods:**

Utilizing a prospective observational approach, we conducted comparative analyses of gut microbiota and metabolites via metabolomics and metagenomics in individuals diagnosed with SA-AKI compared to those without AKI (NCT06197828). Pearson correlations were used to identify associations between microbiota, metabolites, and clinical indicators. The Comprehensive Antibiotic Resistance Database was employed to detect antibiotic resistance genes (ARGs), while Kyoto Encyclopedia of Genes and Genomes (KEGG) pathways informed on metabolic processes and microbial resistance patterns.

**Results:**

Our study included analysis of four patients with SA-AKI and five without AKI. Significant disparities in bacterial composition were observed, illustrated by diversity indices (Shannon index: 2.0 ± 0.4 vs. 1.4 ± 0.6, *P* = 0.230; Simpson index: 0.8 ± 0.1 vs. 0.6 ± 0.2, *P* = 0.494) between the SA-AKI group and the non-AKI group. N6, N6, N6-Trimethyl-L-lysine was detected in both blood and urine metabolites, and also showed significant correlations with specific gut microbiota (Campylobacter hominis and Bacteroides caccae, *R* > 0, *P* < 0.05). Both blood and urine metabolites were enriched in the lysine degradation pathway. We also identified the citrate cycle (TCA cycle) as a KEGG pathway enriched in sets of differentially expressed ARGs in the gut microbiota, which exhibits an association with lysine degradation.

**Conclusions:**

Significant differences in gut microbiota and metabolites were observed between the SA-AKI and non-AKI groups, uncovering potential biomarkers and metabolic changes linked to SA-AKI. The lysine degradation pathway may serve as a crucial link connecting gut microbiota and metabolites.

## 1 Introduction

Sepsis is a life-threatening clinical syndrome characterized by organ dysfunction caused by an abnormal host response to infection (Hotchkiss et al., 2016). Acute kidney injury (AKI) is a common complication of sepsis, and sepsis-associated AKI (SA-AKI) increases the risk of patient mortality (Peng et al., 2014). Existing research suggests that the gut microbiota is involved in the occurrence and progression of SA-AKI (Zhang et al., 2018; Chávez-Iñiguez et al., 2022; Xu et al., 2022). Meijers et al. proposed the concept of the “gut–kidney axis” in 2011; subsequently, Pahl et al. refined the theory (Meijers and Evenepoel, 2011; Pahl and Vaziri, 2015). The core concept of this theory is that kidney injury can disrupt the gut microbiota and impede the functionality of the intestinal epithelial barrier. Dysbiosis in the gut microbiota can also lead to the production of metabolic toxins, thereby exacerbating kidney injury. Wang et al. identified a strong correlation between the composition of the gut microbiota and the serum metabolome in patients with end-stage renal disease (Wang et al., 2020). Moreover, Wu et al. employed dual-omics data to elucidate the intricate links between gut microbiota and perturbed metabolites in the context of chronic kidney disease (CKD) (Wu et al., 2020). Nevertheless, the changes in gut microbiota and metabolites, along with their potential relationship with kidney injury, remain ambiguous in the initial phases of SA-AKI (within the first 48h).

The initial changes in gut microbiota observed in SA-AKI are multifaceted, with the gut microbiota’s influence on the kidneys demonstrating complex and dualistic roles that can lead to both potentially beneficial and detrimental effects (Xu et al., 2022). Alterations in the richness and composition of gut microbiota during the early stages of sepsis have been documented, potentially triggering the onset and progression of AKI (Yu et al., 2018; Chen et al., 2019; Yuan et al., 2020; Xu et al., 2023). These alterations, on one hand, may instigate kidney damage through disruption of the intestinal barrier and amplification of inflammatory responses. Damage to the intestinal mucosal barrier may stimulate pro-inflammatory events, exacerbating the dislocation of gut microbiota and causing kidney damage (Capaldo et al., 2017). Conversely, such modifications may also augment kidney perfusion and oxygenation, thereby bolstering kidney functionality. In 2010, Udy et al. found that patients with severe disease frequently experience hyperfunction in the early stages of the disease (Udy et al., 2010). Gut microbiota’s role in effectively mediating inflammatory responses, which may reduce vascular resistance and increase cardiac output (CO) while affecting capillary permeability; these endogenous reactions combined with aggressive fluid and hemodynamic therapy can further lead to increased kidney blood flow and changes in glomerular filtration (Vaishnavi, 2013; Traykova et al., 2017; Paone and Cani, 2020; Huang et al., 2021). When sepsis occurs, how the gut microbiota affects the kidney and whether it is beneficial or harmful remain unclear.

To elucidate the interaction mechanism and impact of the gut–kidney axis in patients with SA-AKI, we conducted a comparative analysis of the gut microbiota as well as blood and urinary metabolites within 48h of diagnosis in individuals with SA-AKI and those without AKI. Metagenomics and metabolomics approaches were employed for this investigation (ClinicalTrial.gov NCT06197828). Our hypothesis posited that alterations in the gut microbiota during sepsis contribute to changes in the blood metabolome, ultimately exerting comprehensive effects on kidney function through mechanisms such as kidney blood perfusion, oxygen consumption, and inflammatory responses.

## 2 Methods

### 2.1 Participant recruitment and sample collection

**Figure 1:**
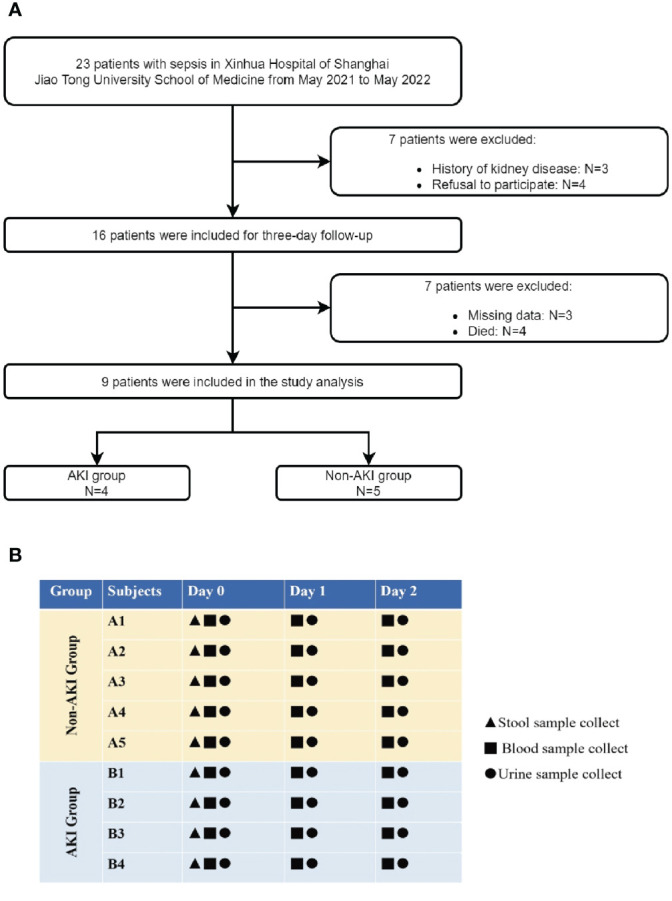
**(A)** Study population. **(B)** Study design and sample collection scheme.

This was a prospective observational study of children admitted to the pediatric intensive care unit (PICU) of Xinhua Hospital, affiliated with the Shanghai Jiao Tong University School of Medicine, from May 2021 to May 2022. The inclusion criteria were as follows: (i) age >28 days and <18 years, and (ii) sepsis diagnosis based on the sepsis-3 criteria (Singer et al., 2016). The exclusion criteria were as follows: (i) history of kidney disease; (ii) no follow-up due to death or discharge within 48h; (iii) refusal to participate in the study by the child’s guardian; and (iv) missing baseline data (**Figure 1A**). The patients were categorized into groups based on the presence or absence of AKI during hospitalization. AKI diagnosis was using Kidney Disease: Improving Global Outcomes (KDIGO) criteria including both plasma creatinine and urine output criteria (Kellum and Lameire, 2013).

After patient enrollment, a 48h follow-up period was conducted. Stool, blood, and urine specimens were collected upon admission [day 0 (D0)], succeeding day 1 (D1) (within the subsequent 24h post admission), as well as on day 2 (D2) (approximately 24h–48h succeeding admission). **Figure 1B** illustrates the study design and the time points for sample collection. This study is reported in concordance with Strengthening the Reporting of Observational Studies in Epidemiology recommendations (von Elm et al., 2007).

The baseline data included age, sex, height, weight, and body mass index at admission. Clinical indicators were assessed during the first 24h in the PICU, including kidney injury markers [serum creatinine (SCr) and blood urea nitrogen (BUN)], hemodynamic parameters [mean arterial pressure (MAP), CO, fractional shortening (FS), ejection fraction (EF), and renal resistive index (RRI)], oxygen metabolism indices (pressure of oxygen in artery (PaO_2_), partial pressure of carbon dioxide in artery (PaCO_2_), saturation of arterial blood oxygen (SaO_2_), lactic acid, pressure of oxygen in urine (PuO_2_), and partial pressure of carbon dioxide in urine (PuCO_2_), and inflammatory indicators (white blood cell, the neutrophil to lymphocyte ratio, IL-8, IL-1β, IL-6, TNF-α, IL-2, IL-10, and procalcitonin). The severity of the disease is evaluated based on the PCI score, PRISM III score, and site of infection. Therapeutic strategies are assessed, which include the administration of antibiotics, vasoactive drugs, mechanical ventilation, and renal replacement therapy. Additionally, the duration of hospitalization and stay in the intensive care unit are also taken into consideration.

The primary objectives were changes in the gut microbiota and blood and urine metabolites between the SA-AKI and non-AKI groups. Secondary objectives included correlations between the gut microbiota or metabolites, clinical indicators, and associations between the gut microbiota and metabolites. Raw data can be accessed from the NCBI BioProject (PRJNA1025259).

### 2.2 Preparation of stool samples and stool metagenomics analysis

Stool microbiome samples were routinely collected on the day of admission using sterile anal swabs and stored at −80°C without preservatives before processing. The Dinfectome Company (Nanjing, China) performed metagenomics next-generation sequencing (NGS) of stool samples as follows: DNA was extracted using the TIANamp Magnetic DNA Kit (Tiangen, Beijing) per the manufacturer’s protocols. RNA was extracted from the bronchoalveolar lavage fluid, plasma, and other samples using the QIAamp Viral RNA Mini Kit (Qiagen, Germany), and a library was constructed after Qubit quantification. DNA libraries were prepared using the Hieff NGS C130P2 OnePot II DNA Library Prep Kit for MGI (Yeasen Biotechnology, Shanghai), per the manufacturer’s protocol. The rRNA was removed from the total RNA, and a library was constructed after purification. Agilent 2100 was used for quality control, and the DNA libraries were 50 bp single-end sequencing on an MGISEQ-200. An in-house bioinformatics pipeline was used for pathogen identification. Briefly, high-quality sequencing data were generated by removing low-quality reads, adapter contamination, and duplicate and short (<36 bp) reads. Human host sequences were identified by mapping to the human reference genome (hs37d5) using the bowtie2 software (version 2.2.6). Gut microbiota data were downloaded from the human gut microbiota database (GMrepo, https://gmrepo.humangut.=home), and all annotated results (Operational Taxonomic Units, OTUs) in our study were compared to public data. Alpha diversity was estimated using Shannon and Simpson indices based on the taxonomic profile of each sample. Differential relative abundance of taxonomic groups at the species level between groups was tested using the Kruskal–Wallis rank sum test (R package kruskal.test). Genera with mean relative abundances >1% and penetrance >40% were compared among all samples. Pearson Correlation coefficients between clinical indicators and the relative abundance of genera were calculated, and false discovery rate correction was used to adjust all *P*-values.

### 2.3 Metabolomics analysis of blood and urine

Venous blood samples were collected through venipuncture in sterile 2 ml of heparinized tubes, and urine samples were collected using sterile tubes (Sterisets Urine Collection Kit). These samples were stored at −20°C before being processed. Liquid chromatography–tandem mass spectrometry (MS) was performed to analyze blood and urine samples (Fujisaka et al., 2018). The profiling procedure included experimental design, quality assurance/control procedures, sampling, metabolite extraction, metabolite measurement, data processing, post-processing, and statistical analysis. Unsupervised principal component analysis (PCA) was used to examine the overall distribution of samples and the stability of the overall analysis process. We used partial least squares discriminant analysis (PLS-DA) and orthogonal PLS-DA (OPLS-DA) to evaluate the differences in metabolic profiles between the SA-AKI and non-AKI groups. Subsequently, differentially represented metabolites were determined based on the value of the variable importance in projection, OPLS-DA model first principal component (threshold >1), and *P*-value of the *t*-test (*P* < 0.05). Progenesis QI (version 2.3, Nonlinear Dynamics, Newcastle, UK) was used for MS analysis. PCA was performed using the R software, and the results were visualized using the ggplot2 package (3.3.6). Heatmaps for visualization were generated using the ComplexHeatmap R package (2.13.1). Pearson’s correlations were calculated to measure the correlation between metabolite levels and clinical indicators. Kyoto Encyclopedia of Genes and Genomes (KEGG) was used to annotate pathways and classify these pathways per the KEGG website (http://www.kegg.jp/) pathway hierarchy classification method.

### 2.4 Analysis of antibiotic resistance genes carriage by gut microbiota

The microbial antibiotic resistance profiles were analyzed utilizing the Comprehensive Antibiotic Resistance Database (CARD). The comparison of antibiotic resistance genes (ARGs) from gut microbiota between groups was performed using DEseq2. The statistical difference was considered significant if the adjusted *P*-value < 0.05 and|log2FC|> 1. Biological processes and KEGG signaling pathways were determined with Metascape.

### 2.5 Statistical analysis

Data were presented as mean ± SD. Comparisons between groups were performed using the nonparametric Kruskal–Wallis test. Statistical analyses were performed using IBM SPSS Statistics for Windows, version 23.0 (IBM Corp., Armonk, NY, USA) and R (version 4.2.1). Statistical significance was set at *P* < 0.05.

## 3 Results

### 3.1 Patient characteristics

Overall, nine patients diagnosed with sepsis, four of whom manifested SA-AKI. Baseline patient characteristics are in **Table 1**. The patients’ mean age was 6.1 years (range, 1–11); seven individuals (77.8%) were men. Among the enrolled participants, 66.7% of patients developed sepsis caused by respiratory tract infections. When comparing the PCI scores and PRISM III scores between the two groups, it was observed that the patients in the SA-AKI group had more severe conditions compared to the non-AKI group.

**Table 1:** Comparisons of baseline characteristics between groups.

	SA-AKI (*n* = 4)	Non-AKI (*n* = 5)
Baseline data		
Age [year, mean (SD)]	8.5 (4.4)	4.2 (3.3)
Gender (male, %)	3 (75.0)	4 (80.0)
Height [m, mean (SD)]	1.2 (0.3)	1.0 (0.3)
Weight [kg, mean (SD)]	25.0 (11.1)	17.7 (10.4)
BMI [kg/m^2^, mean (SD)]	16.4 (2.3)	18.5 (3.0)
Kidney function parameters		
SCr [μmol/L, mean (SD)]	51.2 (17.9)	19.3 (6.6)
BUN [mmol/L, mean (SD)]	9.3 (11.0)	2.8 (1.4)
Hemodynamic parameters		
MAP [mmHg, mean (SD)]	66.3 (3.8)	70.6 (12.1)
CO [L/min, mean (SD)]	3.8 (0.8)	4.1 (2.8)
FS [%, mean (SD)]	28.8 (9.3)	37.8 (2.6)
EF [%, mean (SD)]	55.7 (14.9)	69.3 (3.4)
RRI [mean (SD)]	0.6 (0.1)	0.6 (0.1)
Oxygen metabolism indices		
PaO_2_ [mmHg, mean (SD)]	86.4 (54.9)	71.0 (53.0)
PaCO_2_ [mmHg, mean (SD)]	41.6 (13.6)	37.6 (4.4)
SaO_2_ [%, mean (SD)]	77.6 (25.1)	81.3 (15.6)
Lac [mmol/L, mean (SD)]	1.6 (0.7)	1.5 (0.4)
PuO_2_ [mmHg, mean (SD)]	162.5 (9.6)	117.4 (33.2)
PuCO_2_ [mmHg, mean (SD)]	20.8 (10.4)	39.4 (28.5)
Inflammatory indicators		
WBC [×10^9^/L, mean (SD)]	9.2 (2.5)	13.8 (6.8)
NLR [mean (SD)]	7.9 (3.9)	7.2 (7.3)
IL-8 [pg/ml, mean (SD)]	139.1 (91.5)	1584.3 (3309.7)
IL-1β [pg/ml, mean (SD)]	7.8 (3.5)	84.4 (171.5)
IL-6 [pg/ml, mean (SD)]	41.4 (41.5)	252.0 (430.3)
TNF-α [pg/ml, mean (SD)]	19.3 (6.2)	33.1 (38.6)
IL-2 [U/ml, mean (SD)]	2411.3 (1981.5)	1746.4 (918.1)
IL-10 [pg/ml, mean (SD)]	55.8 (43.8)	13.1 (5.7)
PCT [ng/ml, mean (SD)]	30.0 (46.7)	6.3 (6.3)
Assessment of disease severity		
PCI score [mean (SD)]	87.0 (7.0)	98.4 (2.0)
PRISM III score [mean (SD)]	6.8 (3.3)	0.6 (1.2)
Infection site (*n*, %)		
- Respiratory	3 (75.0)	3 (60.0)
- Blood	1 (25.0)	0
- Central nervous system	0	1 (20.0)
- Multi-site infection	0	1 (20.0)
Treatment		
Antibiotic use (*n*, %)		
- Cephalosporin antibiotics	2 (50.0)	1 (20.0)
- Glycopeptide antibiotics	1 (25.0)	0
- Carbapenem antibiotics	1 (25.0)	0
- Cephamycins antibiotics	0	1 (20.0)
- Combined antibiotics	0	3 (60.0)
Probiotic therapy (*n*, %)	1 (25.0)	2 (40.0)
Vasoactive drugs (*n*, %)	3 (75.0)	1 (20.0)
Mechanical Ventilation (*n*, %)	2 (50.0)	0
RRT (*n*, %)	1 (25.0)	1 (20.0)
Length of hospital stay [day, mean (SD)]	24.8 (10.6)	18.0 (11.7)
Length of ICU stay [day, mean (SD)]	11.5 (5.5)	7.4 (2.1)

SA-AKI, sepsis-induced acute kidney injury; BMI, body mass index; SCr, serum creatinine; BUN, blood urea nitrogen; RRT, renal replacement therapy; ICU, intensive care unit; MAP, mean arterial pressure; CO, cardiac output; FS, fractional shortening; EF, ejection fraction; RRI, renal resistive index; PaO_2_, pressure of oxygen in the artery; PaCO_2_, partial pressure of carbon dioxide in the artery; SaO_2_, saturation of arterial blood oxygen saturation; Lac, lactic acid; PuO_2_, pressure of oxygen in urine; PuCO_2_, partial pressure of carbon dioxide in urine; WBC, white blood cell; NLR, neutrophil-to-lymphocyte ratio; PCT, procalcitonin; PCIS, pediatric critical illness score; PRISM III, pediatric risk of mortality III.

Analysis of clinical indicators in two patient groups. In terms of kidney function indicators, patients with SA-AKI show significantly higher levels of creatinine (51.2 vs. 19.3 μmol/L), along with relatively elevated BUN levels (9.3 vs. 2.8 mmol/L). Hemodynamic analysis reveals that the non-AKI group has superior cardiac function, characterized by comparatively higher MAP, CO, and EF. However, there are no significant differences observed between the two groups in terms of RRI. In terms of oxygen metabolism analysis, the SA-AKI group exhibits higher levels of PaO_2_ (86.4 vs. 71.0) and PuO_2_ (162.5 vs. 117.4) compared to the non-AKI group. Analysis of inflammatory markers shows higher levels of procalcitonin in the SA-AKI group, while the non-AKI group exhibits a more pronounced immune response with higher levels of IL-8, IL-1β, IL-6, and TNF-α.

The usage of antibiotics differed between the SA-AKI and non-AKI groups. Specifically, in the SA-AKI group, 50% of the patients were treated with cephalosporins. Conversely, in the non-AKI group, a combination of third-generation cephalosporins and glycopeptide antibiotics was administered to 60% of the patients. Furthermore, the prevalence of probiotic usage (Clostridium butyricum) was higher in the non-AKI group compared to the SA-AKI group (40% vs. 25%). Patients with SA-AKI had a higher proportion of vasopressor use and mechanical ventilation compared to the non-AKI group. One patient in each group underwent CRRT. The PICU and hospital length of stay were increased in patients with SA-AKI.

### 3.2 Association between gut microbiota and clinical outcomes in patients with sepsis

**Figure 2:**
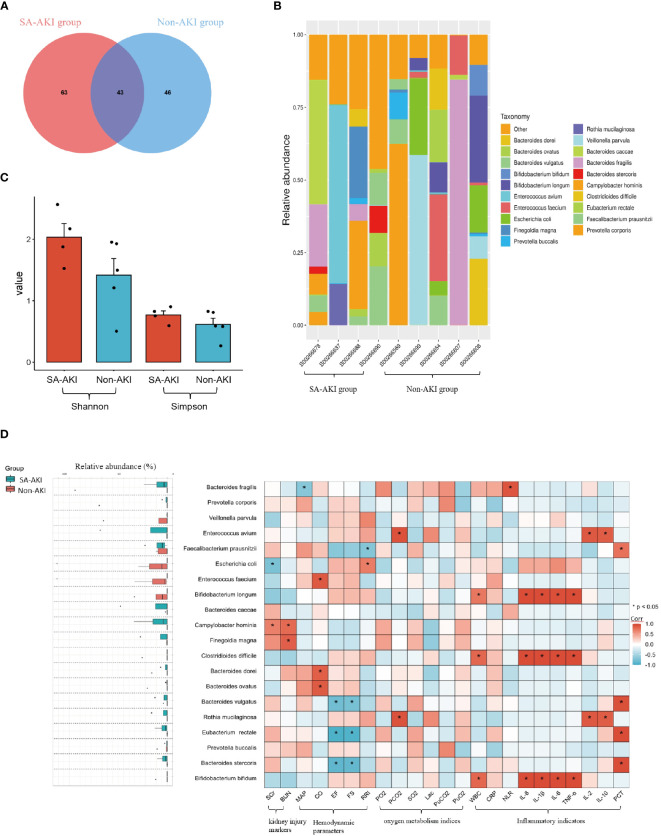
**(A)** Venn representation of gut microbiota; **(B)** stacked bar chart representing the relative abundance at the species level; **(C)** Shannon and Simpson Indices; **(D)** left boxplot showing top 20 relative abundance of gut microbiota at the species level. The right heatmap displays a comparative analysis of gut microbiota between patients’ clinical indicators, and an asterisk indicates microbes significantly interacting in the presence or absence of the factors (Pearson correlation analysis, *P* < 0.05). If the coefficient in the interaction term is >0 (red), the microbe’s abundance increases in the presence of the factor. A value <0 (blue) means the microbe’s abundance decreases in the presence of the factor. AKI, acute kidney injury; SCr, serum creatinine; BUN, blood urea nitrogen; MAP, mean arterial pressure; CO, cardiac output; FS, fractional shortening; EF, ejection fraction; RRI, renal resistive index; PaO_2_, pressure of oxygen in artery; PaCO_2_, partial pressure of carbon dioxide in artery; SaO_2_, saturation of arterial blood oxygen; Lac, lactic acid; PuO_2_, pressure of oxygen in urine; PuCO_2_, partial pressure of carbon dioxide in urine; WBC, white blood cell; NLR, the neutrophil to lymphocyte ratio; PCT, procalcitonin; Corr, correlation.

We performed a comparative analysis of gut microbiota between the SA-AKI group and the non-AKI group. The Venn diagram illustrated that there were 43 OTUs shared at the species level, with a total richness of 152, between the two groups. Additionally, patients in the non-AKI group exhibited a lower number of unique OTUs compared to the SA-AKI group (**Figure 2A**). We conducted a further analysis on the disparities in gut microbiota composition between the SA-AKI group and the non-AKI group. The 20 most abundant gut microbiota at taxonomic levels were chosen to generate a histogram illustrating their relative abundance (**Figure 2B**). At the species level, Enterococcus avium showed the highest abundance in the SA-AKI group, followed by Bacteroides caccae and Campylobacter hominis. In the non-AKI group, the top three species in terms of abundance were Bacteroides fragilis, Veillonella parvula, and Prevotella corporis. The Shannon and Simpson indices displayed higher values in patients diagnosed with SA-AKI as compared to those categorized in the non-AKI group (Shannon index: 2.0 ± 0.4 vs. 1.4 ± 0.6, *P* = 0.230; Simpson index: 0.8 ± 0.1 vs. 0.6 ± 0.2, *P* = 0.494, **Figure 2C**).

Next, we selected the 20 most abundant taxonomic levels of gut microbiota to conduct a correlation analysis with clinical indicators. Regarding kidney function, SA-AKI patients exhibited higher levels of SCr and BUN compared to those without AKI. Additionally, we observed an increase in the expression of Campylobacter hominis in the SA-AKI group. Moreover, we discovered a positive correlation between elevated levels of Campylobacter hominis expression and markers of AKI (SCr, Corr = 0.7, *P* = 0.047; blood urea nitrogen, Corr = 0.8, *P* = 0.012, **Figure 2D**). In relation to cardiovascular function, we found a negative correlation between the levels of the Bacteroidetes (specifically Bacteroides stercoris and Bacteroides vulgatus) and cardiac systolic function (as measured by EF and FS, see **Figure 2D**). Notably, higher levels of the Bacteroidetes were observed in the SA-AKI group. As for the analysis pertaining to oxygen metabolism-related indicators, our study identified an increase in the expression of Rothia mucilaginosa in the SA-AKI group compared to the non-AKI group. This elevation was found to be correlated with higher levels of PaCO_2_ (Corr = 0.9, *P* < 0.001, **Figure 2D**). In relation to inflammatory markers, a notable association was found between the levels of pro-inflammatory cytokines IL-1β, IL-6, IL-8, and TNF-α, and the presence of Bifidobacterium longum, Bifidobacterium bifidum, and Clostridioides difficile. These three types of gut microorganisms exhibited a higher prevalence in the non-AKI group as compared to the SA-AKI group.

The gut microbiota analysis results indicate a significant difference in bacterial composition between the SA-AKI group and the non-AKI group, as evidenced by the distinct relative abundance of predominant taxa. Firmicutes and Bacteroidetes were the dominant phyla in both groups, while Proteobacteria emerged as the third most prevalent phylum in the SA-AKI group. Our clinical relevance analysis further confirms the association of Proteobacteria with AKI markers. Different bacterial species show certain correlations with clinical indicators.

### 3.3 Metabolomics analysis of blood in patients with sepsis

**Figure 3:**
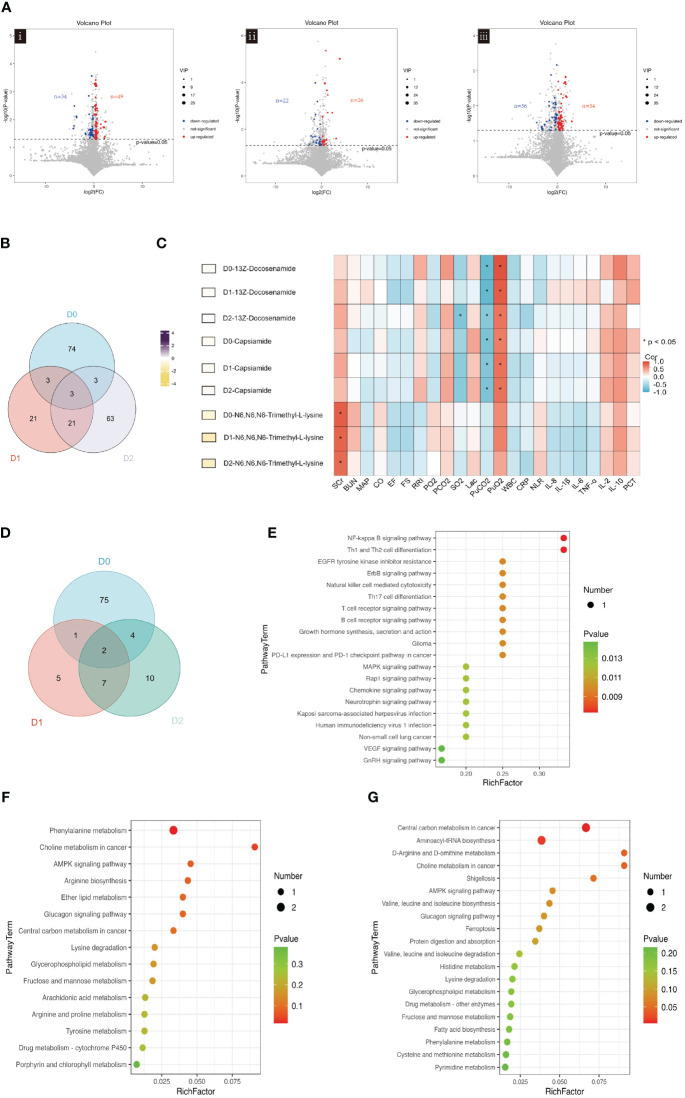
**(A)** Volcano plot showing the differential blood metabolites between SA-AKI and non-AKI groups upon ICU admission (D0, i), at 24h (D1, ii), and at 48 hours (D2, iii). **(B)** Venn diagram illustrating the overlap of blood metabolites in ICU admissions at baseline (D0), 24h after admission (D1), and 48h after admission (D2). **(C)** The left-hand heatmap showing log2 fold change (FC) of expression levels for blood metabolites with coloration ranging from purple (high expression) to yellow (low expression). The closer the color is to bright purple, the higher the expression in the non-AKI group; the closer to bright yellow, the higher the expression in the SA-AKI group. The heatmap on the right displays a correlation using Pearson’s correlation coefficient between blood metabolites and clinical indicators. If the coefficient in the interaction term is >0 (red), the levels of blood metabolite expression increase in the presence of the factor. A value <0 (blue) means a decrease in the expression levels of blood metabolites in the presence of the factor. **(D)** Venn diagrams Illustrates the overlap of significant KEGG pathways identified from the blood metabolites screened in ICU admissions at baseline (D0), 24h post-admission (D1), and 48h post-admission (D2). **(E)** Enrichment analysis of KEGG pathways on D0. **(F)** Enrichment analysis of KEGG pathways on D1. **(G)** Enrichment analysis of KEGG pathways on D2. AKI, acute kidney injury; SCr, serum creatinine; BUN, blood urea nitrogen; MAP, mean arterial pressure; CO, cardiac output; FS, fractional shortening; EF, ejection fraction; RRI, renal resistive index; PaO_2_, pressure of oxygen in artery; PaCO_2_, partial pressure of carbon dioxide in artery; SaO_2_, saturation of arterial blood oxygen; Lac, lactic acid; PuO_2_, pressure of oxygen in urine; PuCO_2_, partial pressure of carbon dioxide in urine; WBC, white blood cell; NLR, the neutrophil to lymphocyte ratio; PCT, procalcitonin; Corr, correlation; ICU, intensive care unit; KEGG, Kyoto Encyclopedia of Genes and Genomes.

We continued the analysis to examine how blood metabolites changed over the first 48h after admission. On D0, 34 metabolites were identified to be downregulated, while 49 metabolites were found to be upregulated (**Figure 3A**i). On D1, 48 differential metabolites were selected, with 22 downregulated and 26 upregulated metabolites observed (**Figure 3A**ii). On D2, 90 differential metabolites were identified, with 36 downregulated and 54 upregulated metabolites observed. (**Figure 3A**iii).

The Venn diagram presented in **Figure 3B** shows the unique and co-differentially expressed metabolites observed in response to AKI at three distinct time points. Specifically, three metabolites, 13Z-docosenamide, Capsiamide, and N6, N6, N6-Trimethyl-L-lysine, exhibited persistence for 48h following admission. Hierarchical clustering was applied to group these metabolites (**Supplementary Figure S1**). Consistently across all three time points, the expression levels of 13Z-docosenamide, Capsiamide, and N6, N6, N6-Trimethyl-L-lysine were found to be higher in the SA-AKI group as compared to the non-AKI group.

The relationship between expression levels of blood metabolite and clinical indicators was explored using Pearson’s correlation analysis. Heatmap analysis was performed to investigate correlations between blood metabolites and clinical indicators (**Supplementary Figure S2**). We identified significant correlations between blood metabolites and clinical indicators: six metabolites correlated with SCr and BUN primarily on D3, 29 with EF and FS mostly on D1, and 15 with PuO_2_ and PuCO_2_. Additionally, 44 metabolites, mainly on D2, were linked to inflammation markers.

**Figure 3C** shows the correlation between clinical indicators and the overlapping blood metabolites, 13Z-docosenamide, Capsiamide, and N6, N6, N6-Trimethyl-L-lysine, at three time points. The levels of these three blood metabolites were significantly higher in the SA-AKI group compared to the non-AKI group. In the first two days, 13Z-docosenamide and Capsiamide exhibited significant positive correlations with PuO_2_, while showing a negative correlation with PuCO_2_. In addition, N6, N6, N6-Trimethyl-L-lysine demonstrated a significant positive correlation with SCr.

The enriched pathways of blood metabolites were analyzed using KEGG enrichment analysis. The Venn diagram the presence of two metabolic pathways, Choline metabolism in cancer and lysine degradation, across all three-time points (**Figure 3D**). It is noteworthy that N6, N6, N6-Trimethyl-L-lysine is involved in the lysine degradation pathways.

On D0, KEGG pathway analysis identified significant enrichment of 68 pathways. Among them, the NF-kappa B signaling pathway and Th1 and Th2 cell differentiation were identified as the two most enriched pathways (**Figure 3E**). The enrichment analysis conducted on D1 revealed a significant enrichment of two KEGG pathways: Phenylalanine metabolism and Choline metabolism in cancer (**Figure 3F**). On D2, four KEGG pathways were found to be significantly enriched: Central carbon metabolism in cancer, Aminoacyl-tRNA biosynthesis, D-arginine, and D-ornithine metabolism, as well as Choline metabolism in cancer (**Figure 3G**).

The findings indicate significant alterations in blood metabolites within the initial 48h, closely linked to clinical indicators. Three metabolites maintained consistent levels during this period. Additionally, two pathways from the KEGG database were persistently active throughout the first two days.

### 3.4 Metabolomics analysis of urine in patients with sepsis

**Figure 4:**
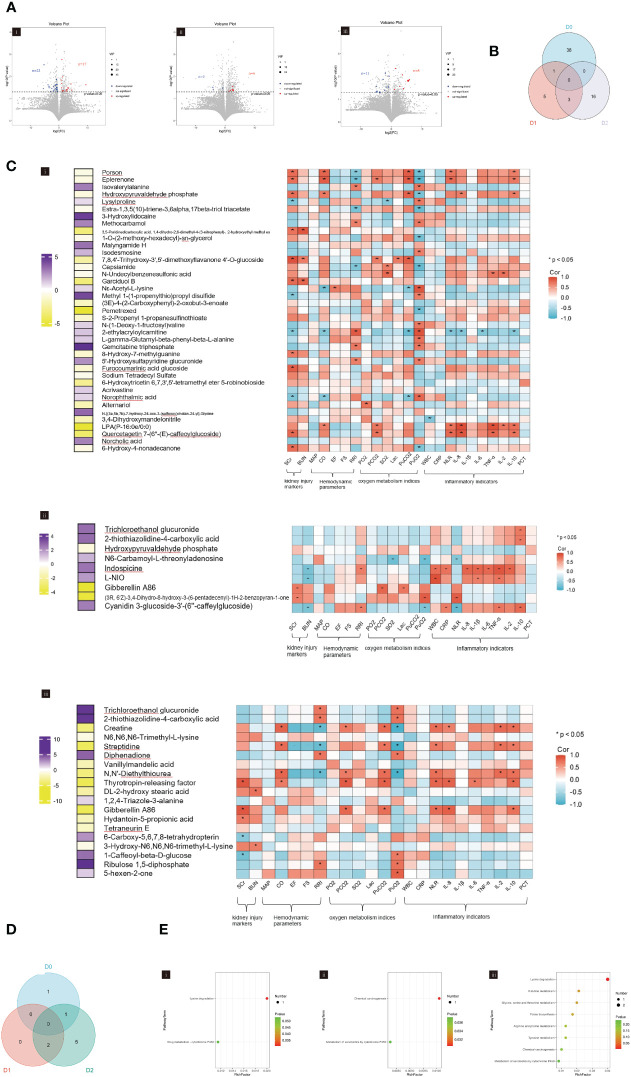
**(A)** Volcano plot showing the differential urinary metabolites between SA-AKI and non-AKI groups upon ICU admission (D0, i), at 24h (D1, ii), and at 48h (D2, iii). **(B)** Venn diagram showing the overlap of urinary metabolites in ICU admissions at baseline (D0), 24h after admission (D1), and 48h after admission (D2). **(C)** The heatmap represents the correlation coefficients between clinical indicators and urinary metabolites upon ICU admission (D0, i), at 24h (D1, ii), and at 48h (D2, iii). The left-hand heatmap showing log_2_ fold change (FC) of expression levels for urinary metabolites with coloration ranging from purple (high expression) to yellow (low expression). The closer the color is to bright purple, the higher the expression in the non-AKI group; the closer to bright yellow, the higher the expression in the SA-AKI group. The heatmap on the right displays a correlation using Pearson’s correlation coefficient between urinary metabolites and clinical indicators. If the coefficient in the interaction term is >0 (red), the expression levels of urinary metabolites increase in the presence of the factor. A value <0 (blue) means a decrease in the expression levels of urinary metabolites in the presence of the factor. **(D)** Venn diagrams illustrate the overlap of significant KEGG pathways identified from the urinary metabolites screened in ICU admissions at baseline (D0), 24h post-admission (D1), and 48h post-admission (D2). **(E)** KEGG pathways enrichment analysis was conducted on ICU admissions at baseline (D0, i), 24h post-admission (D1, ii), and 48h post-admission (D2, iii). AKI, acute kidney injury; SCr, serum creatinine; BUN, blood urea nitrogen; MAP, mean arterial pressure; CO, cardiac output; FS, fractional shortening; EF, ejection fraction; RRI, renal resistive index; PaO_2_, pressure of oxygen in artery; PaCO_2_, partial pressure of carbon dioxide in artery; SaO_2_, saturation of arterial blood oxygen; Lac, lactic acid; PuO_2_, pressure of oxygen in urine; PuCO_2_, partial pressure of carbon dioxide in urine; WBC, white blood cell; NLR, the neutrophil to lymphocyte ratio; PCT, procalcitonin; Corr, correlation; ICU, intensive care unit; KEGG, Kyoto Encyclopedia of Genes and Genomes.

Metabolic profiling of urine showed that a total of 39 differential urinary metabolites were identified on D0 with 22 downregulated and 17 upregulated metabolites (**Figure 4A**i). On D1, nine metabolites were found to be differential, comprising three downregulated and six upregulated metabolites (**Figure 4A**ii). On D2, a total of 19 differential metabolites were identified, including 11 downregulated and eight upregulated metabolites (**Figure 4A**iii).

The Venn diagram depicted in **Figure 4B** illustrates the unique and co-differentially expressed urinary metabolites at three distinct time points. The urinary metabolite, Hydroxypyruvaldehyde phosphate, was detected on both D0 and D1, whereas three urinary metabolites, Trichloroethanol glucuronide, 2-thiothiazolidine-4-carboxylic acid, and Gibberellin A86 remained present on D1 and D2. However, no urinary metabolites persisted across D0, D1, and D2.

Hierarchical clustering was performed to identify clusters (**Supplementary Figure S3**). We have constructed heatmaps to facilitate the visual representation of the correlation coefficients that subsist between clinical indicators and urinary metabolites (**Figure 4C**). In the aspect of kidney injury, 26 (13.7%) urinary metabolites were significantly correlated with SCr and BUN, which were mainly observed on D0 (17, 43.6%). In terms of hemodynamic, three (7.7%) urinary metabolites were observed on D1 and showed increased expression in the SA-AKI group. These metabolites were positively correlated with CO but negatively correlated with RRI. Three (15.8%) urinary metabolites on D2 were also significantly associated with CO and the RRI. Regarding oxygen metabolism, nine (3.1%) urinary metabolites were found to have a significant correlation with PuCO_2_ and PuO_2_ levels, primarily observed on D0 (6, 15.4%). It was observed that the urinary metabolites positively correlated with PuCO_2_ increased in patients with SA-AKI. In the aspect of inflammation, 10 (3.4%) urinary metabolites were significantly associated with indicators of inflammation. Among these, on D0 and D2, they increased in the SA-AKI group and were positively correlated with indicators of inflammation; however, the situation was reversed on D1. The urinary metabolite intersection of D0 and D1, Hydroxypyruvaldehyde phosphate, showed significant associations with SCr, CO, PuCO_2_, PuO_2_, IL-8, and IL-10 levels on D0. However, these correlations were less pronounced on D1. Gibberellin A86, the urinary metabolite intersection of D1 and D2, was found to be significantly associated with SCr and PCO_2_ on both D1 and D2.

The Venn diagram in **Figure 4D** illustrates the intersecting information of the enriched pathways of urinary metabolites at three time points, which were analyzed using KEGG enrichment analysis. There is no overlapping KEGG pathway between D0 and D1. Between D1 and D2, there are two intersecting KEGG pathways, namely the Chemical Carcinogenesis and the Metabolism of Xenobiotics by Cytochrome P450. A sole KEGG pathway, lysine degradation, intersects between D0 and D2. **Figure 4E** presents the enriched pathways of urinary metabolites at three different time points.

The metabolomic analysis of urine reveals significant changes in metabolic profiles at three distinct intervals, with no urinary metabolites demonstrating persistent expression over a 48-hour period. Additionally, no KEGG pathways maintain consistent presence throughout the same timeframe. Notably, while urinary metabolites correlate with clinical indicators, this relation does not remain stable over a prolonged period.

### 3.5 Investigating the potential relationships among gut microbiota, blood metabolites, and urinary metabolites

**Figure 5:**
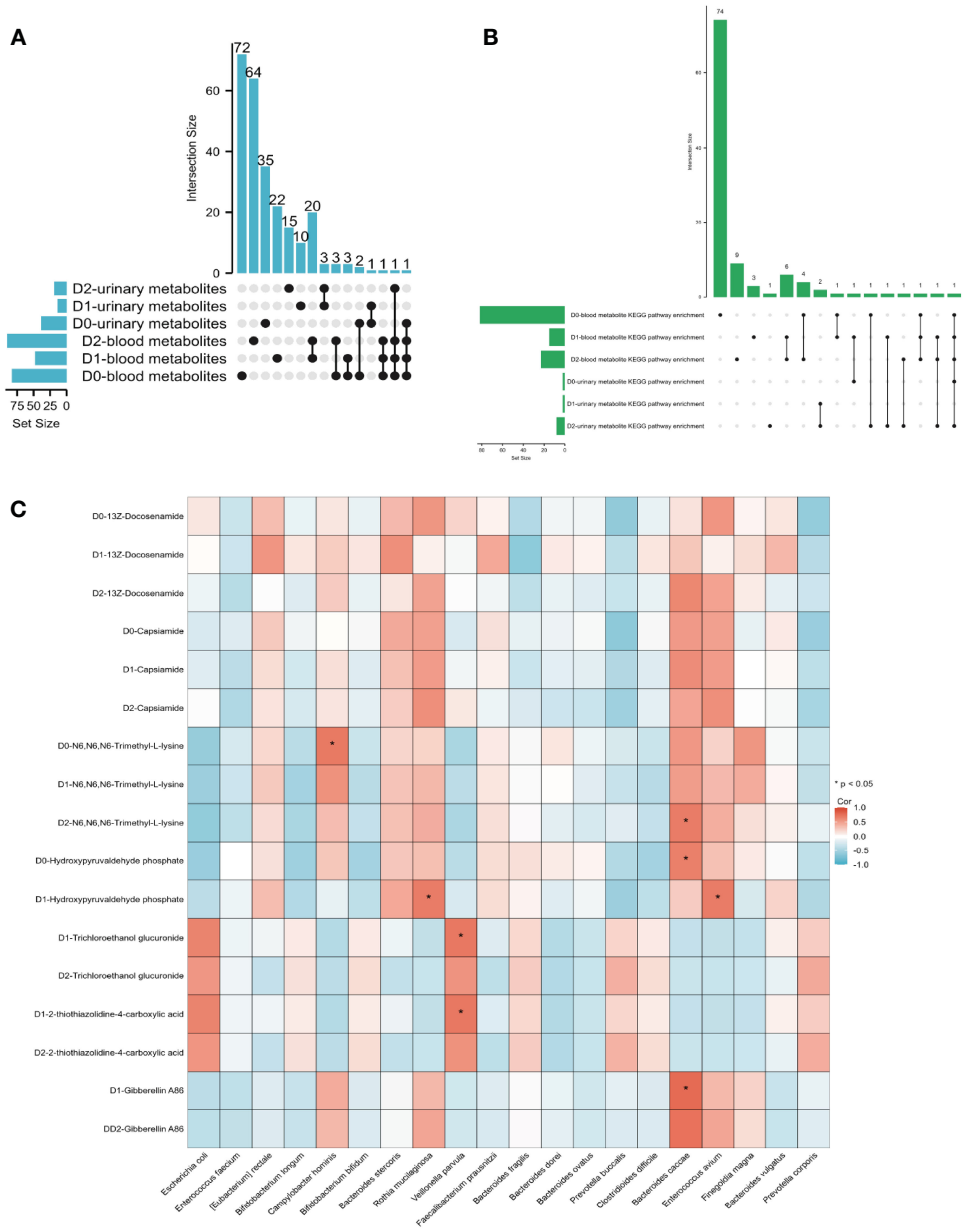
**(A)** The UpSet diagram illustrates the shared metabolites between blood and urine at three-time points. **(B)** The UpSet diagram illustrates the common KEGG enrichment pathway shared by both the blood metabolite KEGG enrichment pathway and the urine metabolite KEGG enrichment pathway at three-time points. **(C)** The heatmap illustrates the Pearson’s correlation between metabolites and gut microbiota. When the coefficient in the interaction term is >0 (depicted in red), it indicates that the expression levels of metabolites increase in the presence of the factor. Conversely, a value <0 (depicted in blue) signifies that the expression levels of metabolites decrease in the presence of the factor.

Existing research has established a close correlation between gut microbiota and metabolites, yet it remains unclear whether this interrelationship is subject to temporal effects (Liu et al., 2022). An UpSet graph was constructed to identify common metabolites between blood metabolites and urinary metabolites at three-time points (**Figure 5A**). Our findings revealed the presence of three metabolites—Porson, Eplerenone, and Capsiamide—in both the blood and urine on D0. In contrast, there were no detectable common metabolites in the blood and urine samples on D1. However, on D2, a congruous metabolite, N6, N6, N6-Trimethyl-L-lysine, was observed in both the blood and urine metabolites.

An UpSet graph was utilized to pinpoint shared KEGG enrichment pathways between both blood and urine metabolite enrichment pathways at three different time points (**Figure 5B**). Our research identified the existence of a single KEGG enrichment pathway, specifically lysine degradation, in both blood and urine samples at D0. Contrarily, there were no observable KEGG enrichment pathways in blood and urine specimens at D1. Nonetheless, at D2, three distinct enrichment pathways, namely, lysine degradation, Histidine metabolism, and Arginine and proline metabolism, were identified in both blood and urine metabolite KEGG enrichment pathways.

We employed Pearson’s correlation analysis to explore the correlation between gut microbiota at D0 and blood metabolites at D0 (A), D1 (B), and D2 (C) (**Supplementary Figure S4**). Additionally, we investigated the relationship between gut microbiota at D0 and urinary metabolites at D0 (A), D1 (B), and D2 (C) using the same analytical approach (**Supplementary Figure S5**). The three blood metabolites, 13Z-Docosenamide, Capsiamide, and N6, N6, N6-Trimethyl-L-lysine, were consistently detected at three different time points. Upon comparing their relationship with the gut microbiota at D0, it was found that there was no significant correlation between the expression of 13Z-Docosanamide and Capsaicamide with the gut microbiota. However, the blood metabolite, N6, N6, N6-Trimethyl-L-lysine, exhibited a notable positive correlation with the gut microbiota, specifically Campylobacter hominis at D0 and Bacteroides caccae at D2 (**Figure 5C**). The urinary metabolite, Hydroxypyruvaldehyde phosphate, which intersects D0 and D1, demonstrated a significant positive association with the gut microbiota Bacteroides caccae on D0, and with Rothia mucilaginosa and Enterococcus avium on D1. Moreover, the intersection of urinary metabolites between D1 and D2, namely, Trichloroethanol glucuronide and 2-thiothiazolidine-4-carboxylic acid, exhibited a substantial positive correlation with Veillonella parvula on D1. Additionally, Gibberellin A86, another commonly detected urinary metabolite in both D1 and D2 samples, displayed a noteworthy positive correlation with Bacteroides caccae on D1 (**Figure 5C**). The fitting curves of the significant correlations between gut microbiota and blood metabolites, as well as urine metabolites, can be found in **Supplementary Figure S6**.

The results suggest an overlap between blood metabolites and urinary metabolites, primarily at D0 and D2. The N6, N6, N6-Trimethyl-L-lysin, which stably exists in the blood metabolites within 48h, is also expressed in D2 urine, and may correlate with specific gut microbiota. Similarly, the KEGG pathway lysine degradation, which is stably present in the blood within 48 hours, is also expressed in D2 urine.

### 3.6 Investigation into the presence of antibiotic resistance genes in the gut microbiota

**Figure 6:**
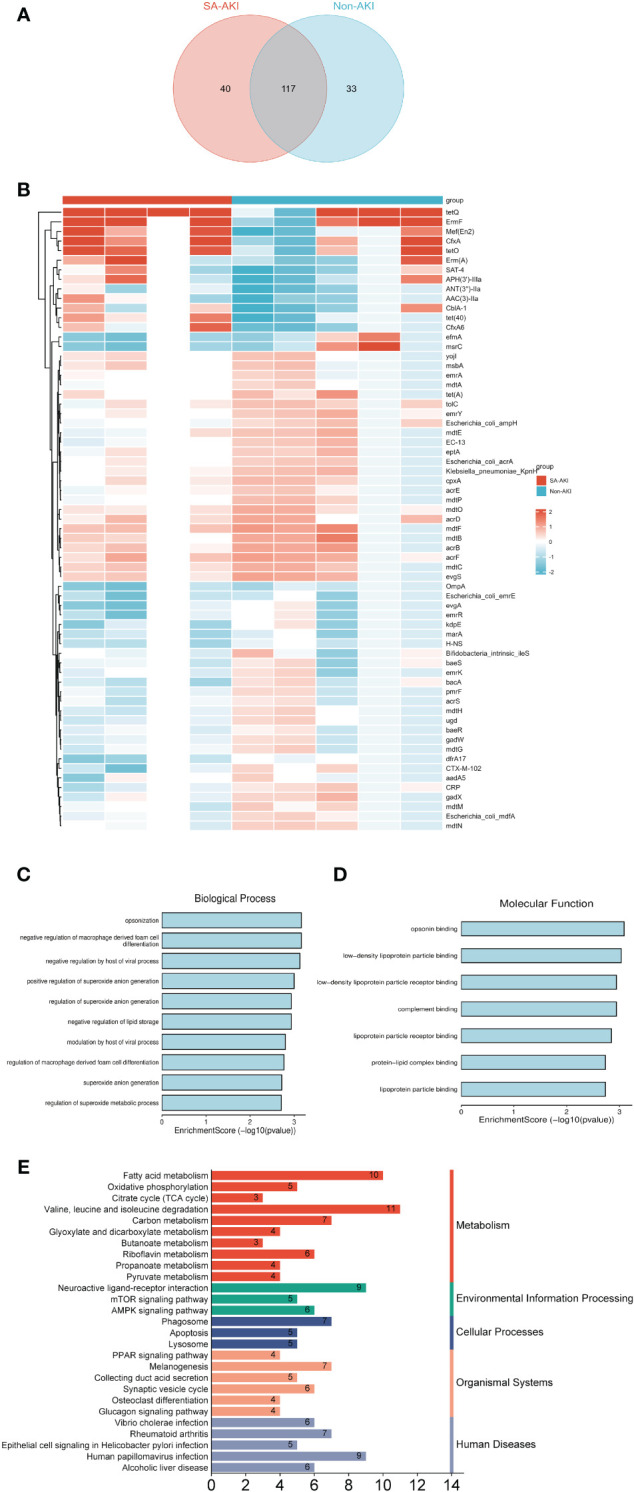
**(A)** The Venn diagram illustrates the presence of antibiotic resistance genes (ARGs) in the gut microbiota of the SA-AKI group and the non-AKI group. **(B)** Heatmap analysis of the number of significantly differentially expressed ARGs. **(C)** Biological process; **(D)** molecular process. **(E)** KEGG pathway enriched in sets of differentially expressed ARGs.

We explored antimicrobial resistance genes in the gut microbiota utilizing the CARD. The Venn diagram in **Figure 6A** illustrates the detection of 157 ARGs in the gut microbiota of the SA-AKI group and 150 ARGs in the non-AKI group, with 117 genes common to both groups. We identified 65 significantly differentially expressed ARGs based on our screening criteria (adjusted *P* < 0.05 and |log2FC| > 1). **Figure 6B** illustrates the heatmap of the number of significantly differentially expressed ARGs. We conducted a comparative analysis of the expression of GO entries with a *P* < 0.05. The primary biological processes identified included Opsonization, Negative regulation of macrophage-derived foam cell differentiation, and Negative regulation by the host of viral processes (**Figure 6C**). In terms of molecular function, the most significant enrichment and meaningful terms are opsonin binding, suggesting a functional role at the molecular level (**Figure 6D**). However, no significant enrichment was observed in terms related to cellular components. We identified KEGG pathway enriched in sets of differentially expressed ARGs using Metascape. The top KEGG terms included “Valine, leucine and isoleucine degradation,” “Fatty acid metabolism,” “Human papillomavirus infection” and “Neuroactive ligand-receptor interaction” (**Figure 6E**). It merits a particular emphasis that the lysine degradation, a KEGG pathway common to both blood and urinary metabolites, exhibits an association with the citrate cycle (TCA cycle). **Supplementary Figure S7** displays an UpSet diagram illustrating the shared KEGG enrichment pathways among blood metabolites, urine metabolites, and antibiotic-resistance genes in the gut microbiota. KEGG pathway analysis shows no overlap between urinary metabolites and gut microbiota ARGs. On D0, blood metabolites and ARGs overlap in Melanogenesis and Vibrio cholerae infection pathways. By days 1 and 2, overlaps include the AMPK and Glucagon signaling pathways, with day 2 adding Valine, leucine, and isoleucine degradation. The interaction among KEGG pathways suggests a potential correlation between blood and urinary metabolites and ARGs in the gut microbiota.

## 4 Discussion

To clarify the interaction mechanism and influence of the gut–kidney axis in patients with SA-AKI, we conducted a comparative analysis of gut microbiota as well as blood and urinary metabolites between individuals with SA-AKI and those without AKI using metagenomics and metabolomics approaches.

There is a decrease in the abundance and diversity of gut microbiota in patients with sepsis (Zhu et al., 2018; Chen et al., 2019). However, we observed an increase in the diversity of gut microbiota in patients with SA-AKI increased. Similar results were also reported in Yang et al.’s study, suggesting that kidney injury can profoundly alter the composition of gut microbiota, leading to distinct microbial community profiles (Yang et al., 2020). We observed the absence of beneficial anaerobes, such as Bifidobacterium, in patients with SA-AKI, while the expression of Enterobacter was found to be elevated. Dicu-Andreescu et al. and Nakade et al. have reported similar findings (Nakade et al., 2018; Dicu-Andreescu et al., 2024). The most notable characteristic of the alteration in the gut microbiota composition in sepsis patients is the reduction in protective symbiotic flora, specifically obligate anaerobic bacteria and Lactobacilli, alongside the elevation of pathogenic bacteria such as Enterococcus and Pseudomonas, which can become predominant (Dickson, 2016; Lankelma et al., 2017; Wan et al., 2018).

We have also discussed the correlation between alterations in gut microbiota and clinical indicators in SA-AKI patients. Multiple mechanisms, including macrovascular and microvascular dysfunction, inflammatory cascades, and kidney oxygen supply, mediate SA-AKI (Peerapornratana et al., 2019; Chang et al., 2022). Here, we found that both gut microbiota and metabolites were involved in these mechanisms. We have observed an interesting phenomenon: the inflammatory response of patients in the non-AKI group appears to be more pronounced compared to that of the AKI group. It is worth noting that the non-AKI group, despite the heightened inflammatory response, exhibits higher MAP, CO, and EF values when compared to the SA-AKI group. This finding seems to contradict the results of certain studies that indicate inflammatory responses may worsen kidney damage. This appears to contradict some studies’ findings, suggesting that inflammatory responses can exacerbate kidney damage (Bijuklic et al., 2007; Andres-Hernando et al., 2012). This phenomenon may be related to the presence of systemic inflammatory response syndrome, which leads to augmented renal clearance. Early stages of sepsis are accompanied by the release of pro-inflammatory mediators, which may reduce vascular resistance and increase CO while affecting capillary permeability (Langenberg et al., 2005; Trof et al., 2010). Here, we found that an elevation in the levels of Bifidobacterium longum, Bifidobacterium bifidum, and Clostridium difficile in the non-AKI group, and these changes were found to be closely associated with inflammatory factors. This observation is consistent with findings from other relevant studies (Bassotti et al., 2023). We have also identified a relationship between Enterococcus faecium and Bacteroides stercoris, as well as Bacteroides vulgatus. Studies have emphasized that it may be related to cardiac dysfunction and vascular permeability changes caused by systemic inflammation in patients with sepsis. The complex alterations in gut microbiota during SA-AKI could potentially exert a multitude of interactive impacts on kidney function (Rabkin, 2009).

The existing research has demonstrated a robust correlation between the gut microbiota and blood metabolites, a relationship that our study also confirmed (Zhang et al., 2020). Here, we found that three metabolites (13Z-docosenamide, capsiamide, and N6, N6, N6-Trimethyl-L-lysine) in patients with SA-AKI persisted at the three-time points and were closely related to the gut microbiota. Reportedly, the production of 13Z-docosenamide by Escherichia coli and similar or related molecules by other bacteria is crucial during growth (Tamilmani et al., 2018). Some studies have suggested that 13Z-docosenamide serves as the primary angiogenic lipid in bovine mesentery, as demonstrated through a chorioallantoic membrane assay (Wakamatsu et al., 1990; Mitchell et al., 1996). Zhou et al. found that Capsiamide is linked to Clostridium difficile and may act as an anti-inflammatory (Zhou et al., 2018). N6, N6, N6-trimethyl-L-lysine are positively associated with Phascola crobacterium, which is involved in regulating water uptake (Li et al., 2023). These studies suggest that the gut microbiota plays a vital role in nutrient digestion and absorption and influences blood metabolism (Turnbaugh and Gordon, 2008; Org et al., 2017). Visconti et al. observed approximately seven times more associations between metabolites and microbial metabolic pathways compared to species. The trend became even more pronounced r when analyzing the fecal–blood dialog, with approximately 13 times more co-associated metabolite pairs identified using the P-gain statistics for microbial metabolic pathways compared to species (Visconti et al., 2019). Therefore, this could be a key point in our research to make the gut–kidney axis possible. A joint study of the microbiome and metabolome has been suggested as the most promising approach for evaluating host–microbiome interactions.

Here, simultaneous changes in the blood and urinary metabolites of patients with SA-AKI were analyzed. The blood metabolome undergoes profound changes in response to kidney injury, whereas the urine metabolome is expected to be directly related to the kidney because of its convenience (Kalantari and Nafar, 2019). A study analyzed urine metabolites in patients with AKI after cardiac surgery with cardiopulmonary bypass and showed that the spectra of 24h postoperative urine specimens could predict AKI across all stages with an average accuracy of 76.0% and a corresponding AUC value of 0.83 (Zacharias et al., 2013). In our study, however, urinary metabolites were found to be unstable, with no consistently expressed urinary metabolites across three-time points or enriched KEGG pathways identified. It is important to highlight that N6, N6, N6-Trimethyl-L-lysine was detected in blood metabolites at three time points and in urine metabolites on D2. Additionally, both blood and urinary metabolites demonstrated an enhancement in the lysine degradation pathway. This pathway is fundamental to cellular growth, differentiation, and metabolic regulation, with N6, N6, N6-Trimethyl-L-lysine also featuring within the process (Feng et al., 2022; Yuan et al., 2023).

The gut serves as a crucial harborage for drug-resistant bacteria. A robust gut microbiota, characterized by stability and diversity, safeguards the host against encroachment by pathogenic bacteria. Antibiotic treatments can potentially destabilize the gut’s harmonious ecosystem, thereby creating a conducive environment for drug-resistant bacteria colonization. This situation escalates the load of resistance genes and exacerbates the dispersion of resistant bacteria to other areas, leading to infections (Anthony et al., 2021). Similarly, antibiotic usage can affect microbiota stability (Chávez-Íñiguez et al., 2023). In this study, all patients had undergone antibiotic treatment, with 33.3% having utilized probiotics. We have assessed whether the enriched KEGG pathways of these ARGs correspond to certain sections relating to blood and urinary metabolites. The lysine degradation pathway, a KEGG pathway concurrently observed in blood and urinary metabolites, demonstrates a significant correlation with the citrate cycle (TCA cycle). The existing research confirms that lysine acetylation/deacetylation plays a crucial role in regulating metabolic enzymes in the TCA cycle, primarily through the functions of sirtuins (Shakespear et al., 2018). Moreover, essential metabolic enzymes and energy metabolites have a direct influence on the pro-inflammatory and anti-inflammatory responses of macrophages. These findings may contribute to our clinically relevant markers, although additional research is needed to validate this hypothesis.

This study had some limitations. First, a small sample size and insufficient postoperative follow-up time. Although the sample size of this study met the requirements of a randomized controlled trial, the relatively small sample size may have led to a certain deviation in the results; therefore, follow-up studies with larger sample sizes are required to validate these findings. Second, due to constraints in sample availability and funding, we were unable to perform multivariate analysis on the gut microbiota at time points D1 and D2. This may affect the accuracy of our results. However, in practice, it is often impractical to analyze patients’ samples over an extended period due to substantial financial requirements, resulting in a predominant preference for data from D0. Hence, exploring the data from D0 and the persistent expression of metabolites in this study could prove beneficial for clinical settings. Third, in this study, our primary focus is on observation and demonstration. We explore the correlation between gut microbiota and blood and urinary metabolites, with the aim of identifying breakthrough points in the gut–kidney axis. As a result, we have not further corroborated the related metabolic products, genes, and KEGG pathways found in the research. However, we plan to validate these findings through animal and cellular experiments in subsequent studies.

## 5 Conclusion

In conclusion, significant differences were noted in gut microbiota and both blood and urinary metabolites between the SA-AKI and non-AKI groups. These differences showed varying correlations with clinical indicators over time. The blood metabolites, 13Z-docosenamide, capsiamide, and N6, N6, N6-trimethyl-L-lysine, were detected at all three time points, whereas no consistently expressed metabolites were found in the urinary samples at all three time points. N6, N6, N6-Trimethyl-L-lysine, present in both blood and urine, was enriched in the lysine degradation pathway, a common KEGG pathway. Additionally, the study identified ARGs in the population, with several correlating with blood metabolites, notably the citrate cycle (TCA cycle) linked to lysine degradation.

## Data availability statement

Raw data can be accessed from the NCBI BioProject (PRJNA1025259). Other data supporting the findings of this study are available from the corresponding author upon reasonable request.

## Ethics statement

This study was approved by the Ethics Committee of Xinhua Hospital, Shanghai Jiao Tong University School of Medicine (approval number: XHEC-D-2022-255). This is a prospective observational study and has been registered with ClinicalTrials.gov (NCT06197828). The studies were conducted in accordance with the local legislation and institutional requirements. Written informed consent for participation in this study was provided by the participants’ legal guardians/next of kin.

## Author contributions

YX: Writing – original draft, Writing – review & editing, Conceptualization, Data curation, Formal analysis, Funding acquisition, Investigation, Methodology, Project administration, Resources, Software. JX: Data curation, Formal analysis, Investigation, Methodology, Writing – original draft. YZ: Project administration, Resources, Visualization, Writing – original draft. HM: Data curation, Methodology, Writing – original draft. JL: Formal analysis, Project administration, Supervision, Writing – original draft. XK: Data curation, Formal analysis, Methodology, Writing – original draft. XZ: Supervision, Validation, Writing – review & editing. JZ: Formal analysis, Project administration, Supervision, Writing – review & editing.
