# Supplementary material for: Investigating gut microbiota–blood and urine metabolite correlations in early sepsis-induced acute kidney injury: insights from targeted KEGG analyses
Source: Front Cell Infect Microbiol. 2024 Jun 3;14:1375874. doi: 10.3389/fcimb.2024.1375874 (PMC11180806; doi:10.3389/fcimb.2024.1375874)
Supplement: Supplementary file 1 [file DataSheet_1.pdf]

Figure S1 A

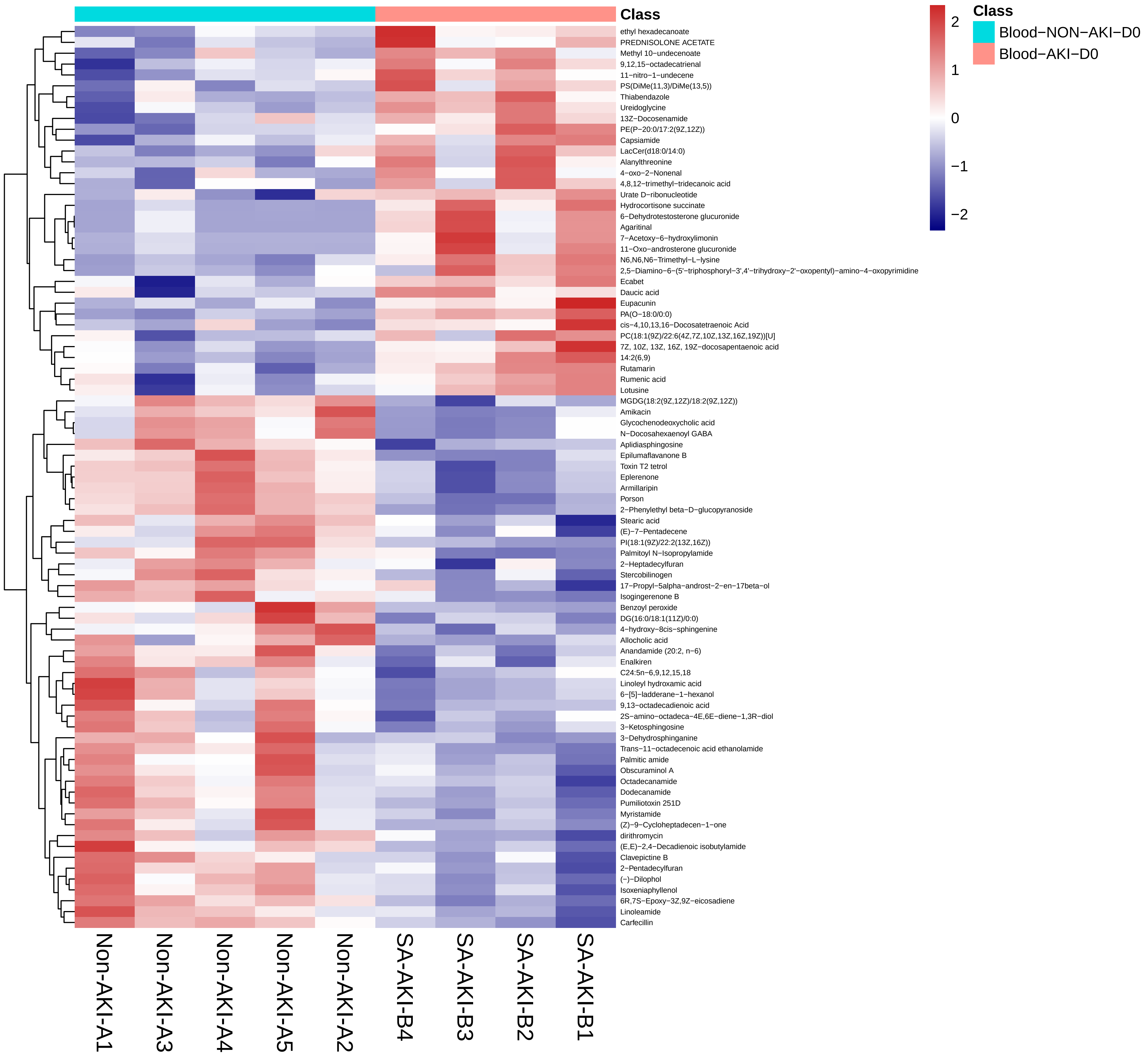

Class
Class
Blood−NON−AKI−D0
Blood−AKI−D0
2
1
0
−1
−2
ethyl hexadecanoate
PREDNISOLONE ACETATE
Methyl 10−undecenoate
9,12,15−octadecatrienal
11−nitro−1−undecene
PS(DiMe(11,3)/DiMe(13,5))
Thiabendazole
Ureidoglycine
13Z−Docosenamide
PE(P−20:0/17:2(9Z,12Z))
Capsiamide
LacCer(d18:0/14:0)
Alanylthreonine
4−oxo−2−Nonenal
4,8,12−trimethyl−tridecanoic acid
Urate D−ribonucleotide
Hydrocortisone succinate
6−Dehydrotestosterone glucuronide
Agaritinal
7−Acetoxy−6−hydroxylimonin
11−Oxo−androsterone glucuronide
N6,N6,N6−Trimethyl−L−lysine
2,5−Diamino−6−(5'−triphosphoryl−3',4'−trihydroxy−2'−oxopentyl)−amino−4−oxopyrimidine
Ecabet
Daucic acid
Eupacunin
PA(O−18:0/0:0)
cis−4,10,13,16−Docosatetraenoic Acid
PC(18:1(9Z)/22:6(4Z,7Z,10Z,13Z,16Z,19Z))[U]
7Z, 10Z, 13Z, 16Z, 19Z−docosapentaenoic acid
14:2(6,9)
Rutamarin
Rumenic acid
Lotusine
MGDG(18:2(9Z,12Z)/18:2(9Z,12Z))
Amikacin
Glycochenodeoxycholic acid
N−Docosahexaenoyl GABA
Aplidiasphingosine
Epilumaflavanone B
Toxin T2 tetrol
Eplerenone
Armillaripin
Porson
2−Phenylethyl beta−D−glucopyranoside
Stearic acid
(E)−7−Pentadecene
PI(18:1(9Z)/22:2(13Z,16Z))
Palmitoyl N−Isopropylamide
2−Heptadecylfuran
Stercobilinogen
17−Propyl−5alpha−androst−2−en−17beta−ol
Isogingerenone B
Benzoyl peroxide
DG(16:0/18:1(11Z)/0:0)
4−hydroxy−8cis−sphingenine
Allocholic acid
Anandamide (20:2, n−6)
Enalkiren
C24:5n−6,9,12,15,18
Linoleyl hydroxamic acid
6−[5]−ladderane−1−hexanol
9,13−octadecadienoic acid
2S−amino−octadeca−4E,6E−diene−1,3R−diol
3−Ketosphingosine
3−Dehydrosphinganine
Trans−11−octadecenoic acid ethanolamide
Palmitic amide
Obscuraminol A
Octadecanamide
Dodecanamide
Pumiliotoxin 251D
Myristamide
(Z)−9−Cycloheptadecen−1−one
dirithromycin
(E,E)−2,4−Decadienoic isobutylamide
Clavepictine B
2−Pentadecylfuran
(−)−Dilophol
Isoxeniaphyllenol
6R,7S−Epoxy−3Z,9Z−eicosadiene
Linoleamide
Carfecillin
Non-AKI-A1
Non-AKI-A3
Non-AKI-A4
Non-AKI-A5
Non-AKI-A2
SA-AKI-B4
SA-AKI-B3
SA-AKI-B2
SA-AKI-B1

Figure S1 B

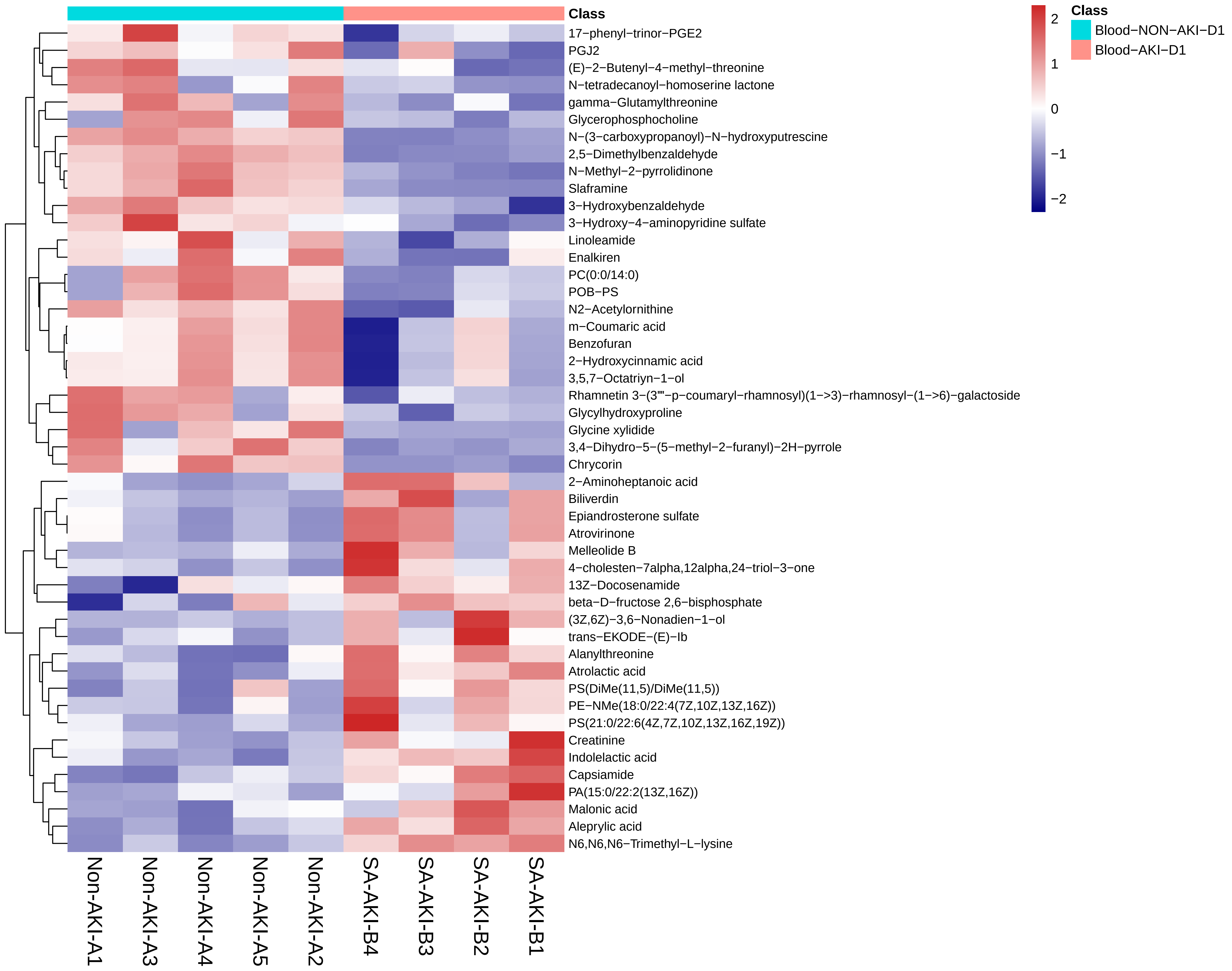

Class
17−phenyl−trinor−PGE2
PGJ2
(E)−2−Butenyl−4−methyl−threonine
N−tetradecanoyl−homoserine lactone
gamma−Glutamylthreonine
Glycerophosphocholine
N−(3−carboxypropanoyl)−N−hydroxyputrescine
2,5−Dimethylbenzaldehyde
N−Methyl−2−pyrrolidinone
Slaframine
3−Hydroxybenzaldehyde
3−Hydroxy−4−aminopyridine sulfate
Linoleamide
Enalkiren
PC(0:0/14:0)
POB−PS
N2−Acetylornithine
m−Coumaric acid
Benzofuran
2−Hydroxycinnamic acid
3,5,7−Octatriyn−1−ol
Rhamnetin 3−(3'''−p−coumaryl−rhamnosyl)(1−>3)−rhamnosyl−(1−>6)−galactoside
Glycylhydroxyproline
Glycine xylidide
3,4−Dihydro−5−(5−methyl−2−furanyl)−2H−pyrrole
Chrycorin
2−Aminoheptanoic acid
Biliverdin
Epiandrosterone sulfate
Atrovirinone
Melleolide B
4−cholesten−7alpha,12alpha,24−triol−3−one
13Z−Docosenamide
beta−D−fructose 2,6−bisphosphate
(3Z,6Z)−3,6−Nonadien−1−ol
trans−EKODE−(E)−Ib
Alanylthreonine
Atrolactic acid
PS(DiMe(11,5)/DiMe(11,5))
PE−NMe(18:0/22:4(7Z,10Z,13Z,16Z))
PS(21:0/22:6(4Z,7Z,10Z,13Z,16Z,19Z))
Creatinine
Indolelactic acid
Capsiamide
PA(15:0/22:2(13Z,16Z))
Malonic acid
Aleprylic acid
N6,N6,N6−Trimethyl−L−lysine
Non-AKI-A1
Non-AKI-A3
Non-AKI-A4
Non-AKI-A5
Non-AKI-A2
SA-AKI-B4
SA-AKI-B3
SA-AKI-B2
SA-AKI-B1
2
1
0
−1
−2
Class
Blood−NON−AKI−D1
Blood−AKI−D1

Figure S1 C

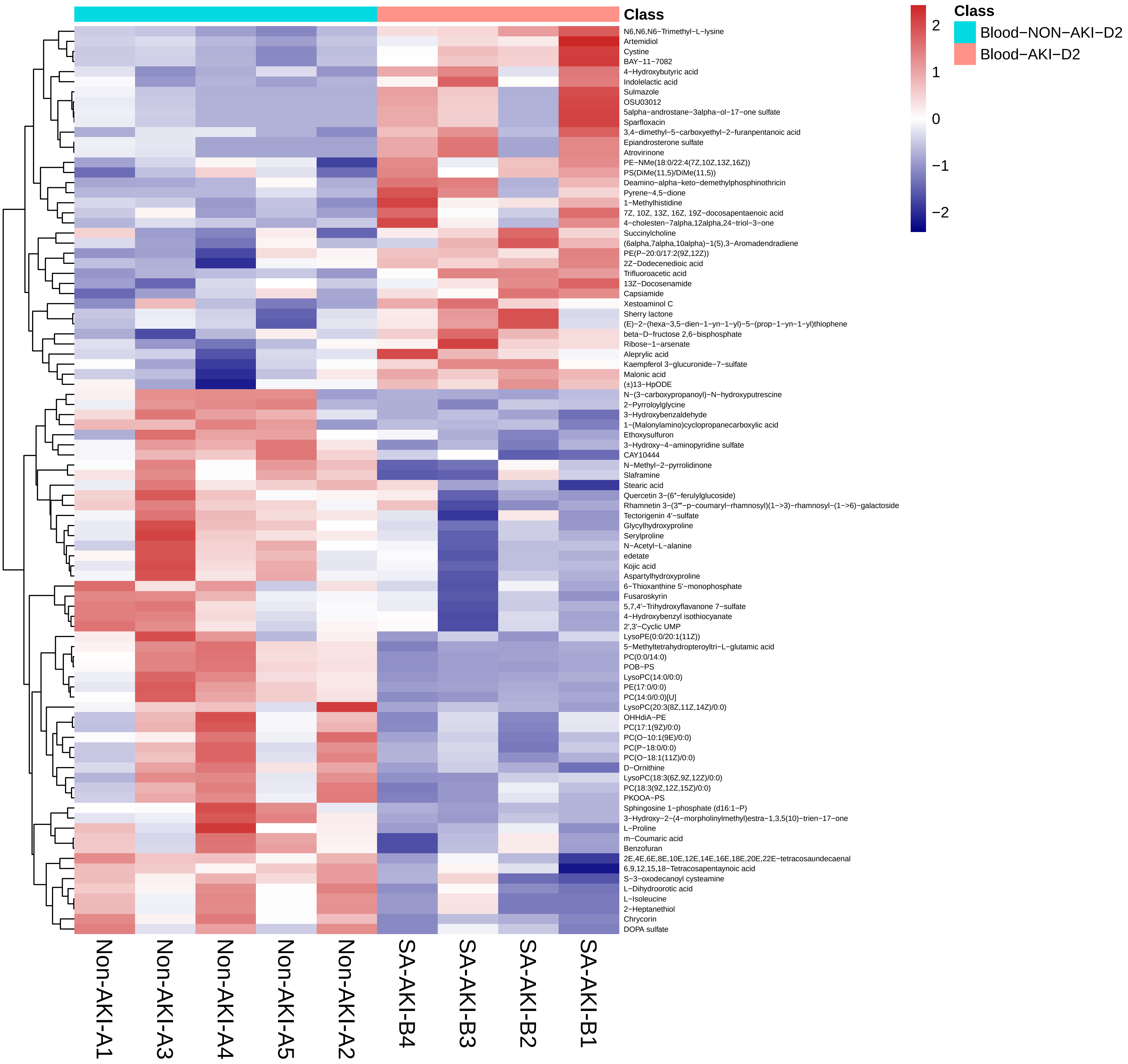

Figure S1 Heatmap showing hierarchical clustering of the blood metabolite enrichment analysis at D0 (A), D1(B) and D2 (C).
