# Supplementary material for: Investigating gut microbiota–blood and urine metabolite correlations in early sepsis-induced acute kidney injury: insights from targeted KEGG analyses
Source: Front Cell Infect Microbiol. 2024 Jun 3;14:1375874. doi: 10.3389/fcimb.2024.1375874 (PMC11180806; doi:10.3389/fcimb.2024.1375874)
Supplement: Supplementary file 2 [file DataSheet_2.pdf]

Figure S2 A

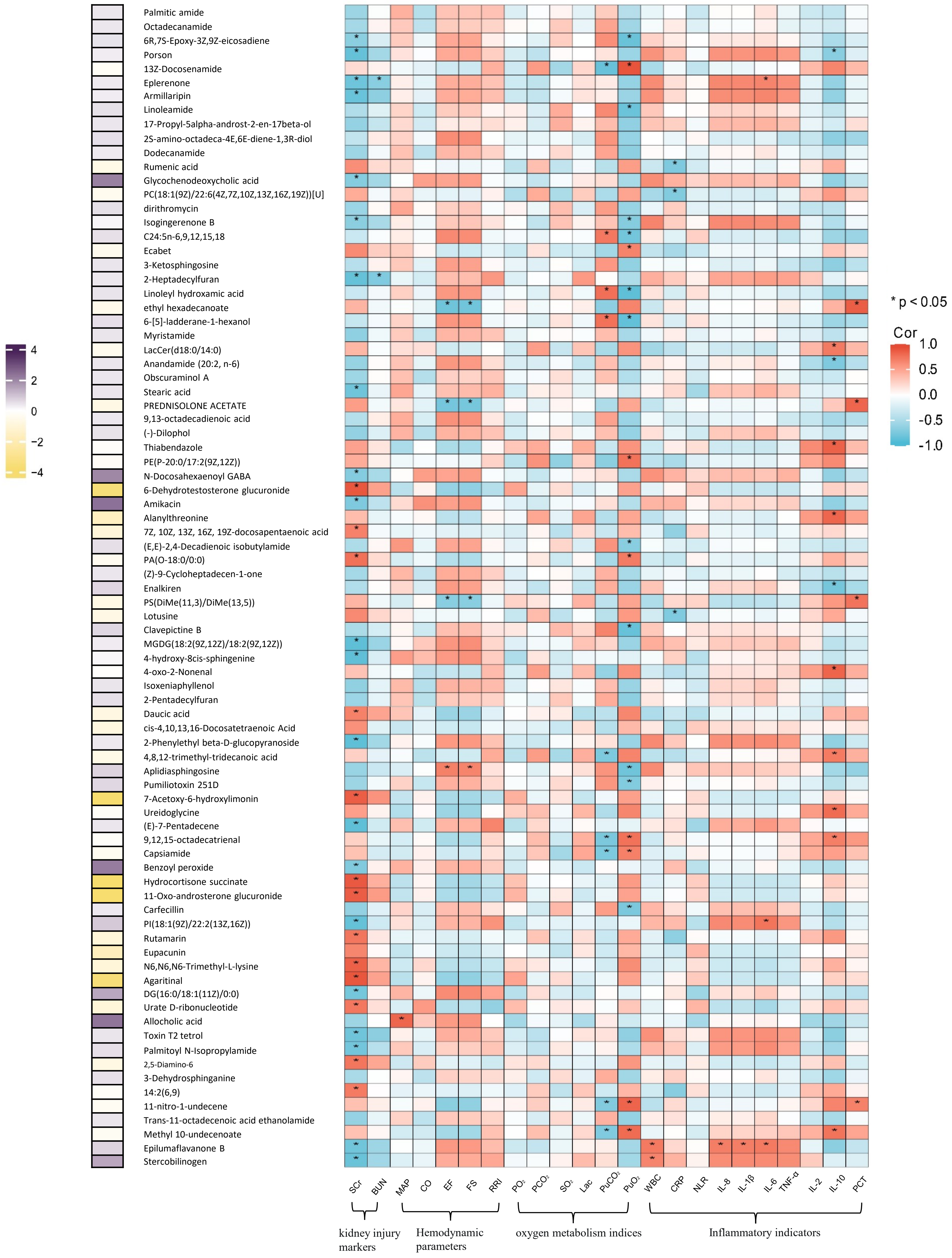

4
2
0
−2
−4
Palmitic amide
Octadecanamide
6R,7S-Epoxy-3Z,9Z-eicosadiene
Porson
13Z-Docosenamide
Eplerenone
Armillaripin
Linoleamide
17-Propyl-5alpha-androst-2-en-17beta-ol
2S-amino-octadeca-4E,6E-diene-1,3R-diol
Dodecanamide
Rumenic acid
Glycochenodeoxycholic acid
PC(18:1(9Z)/22:6(4Z,7Z,10Z,13Z,16Z,19Z))[U]
dirithromycin
Isogingerenone B
C24:5n-6,9,12,15,18
Ecabet
3-Ketosphingosine
2-Heptadecylfuran
Linoleyl hydroxamic acid
ethyl hexadecanoate
6-[5]-ladderane-1-hexanol
Myristamide
LacCer(d18:0/14:0)
Anandamide (20:2, n-6)
Obscuraminol A
Stearic acid
PREDNISOLONE ACETATE
9,13-octadecadienoic acid
(-)-Dilophol
Thiabendazole
PE(P-20:0/17:2(9Z,12Z))
N-Docosahexaenoyl GABA
6-Dehydrotestosterone glucuronide
Amikacin
Alanylthreonine
7Z, 10Z, 13Z, 16Z, 19Z-docosapentaenoic acid
(E,E)-2,4-Decadienoic isobutylamide
PA(O-18:0/0:0)
(Z)-9-Cycloheptadecen-1-one
Enalkiren
PS(DiMe(11,3)/DiMe(13,5))
Lotusine
Clavepictine B
MGDG(18:2(9Z,12Z)/18:2(9Z,12Z))
4-hydroxy-8cis-sphingenine
4-oxo-2-Nonenal
Isoxeniaphyllenol
2-Pentadecylfuran
Daucic acid
cis-4,10,13,16-Docosatetraenoic Acid
2-Phenylethyl beta-D-glucopyranoside
4,8,12-trimethyl-tridecanoic acid
Aplidiasphingosine
Pumiliotoxin 251D
7-Acetoxy-6-hydroxylimonin
Ureidoglycine
(E)-7-Pentadecene
9,12,15-octadecatrienal
Capsiamide
Benzoyl peroxide
Hydrocortisone succinate
11-Oxo-androsterone glucuronide
Carfecillin
PI(18:1(9Z)/22:2(13Z,16Z))
Rutamarin
Eupacunin
N6,N6,N6-Trimethyl-L-lysine
Agaritinal
DG(16:0/18:1(11Z)/0:0)
Urate D-ribonucleotide
Allocholic acid
Toxin T2 tetrol
Palmitoyl N-Isopropylamide
2,5-Diamino-6
3-Dehydrosphinganine
14:2(6,9)
11-nitro-1-undecene
Trans-11-octadecenoic acid ethanolamide
Methyl 10-undecenoate
Epilumaflavanone B
Stercobilinogen
SCr
BUN
MAP
CO
EF
FS
RRI
PO₂
PCO₂
SO₂
Lac
PuCO₂
PuO₂
WBC
CRP
NLR
IL-8
IL-1β
IL-6
TNF-α
IL-2
IL-10
PCT
kidney injury markers
Hemodynamic parameters
oxygen metabolism indices
Inflammatory indicators
* p < 0.05
Cor
1.0
0.5
0.0
-0.5
-1.0

Figure S2 B

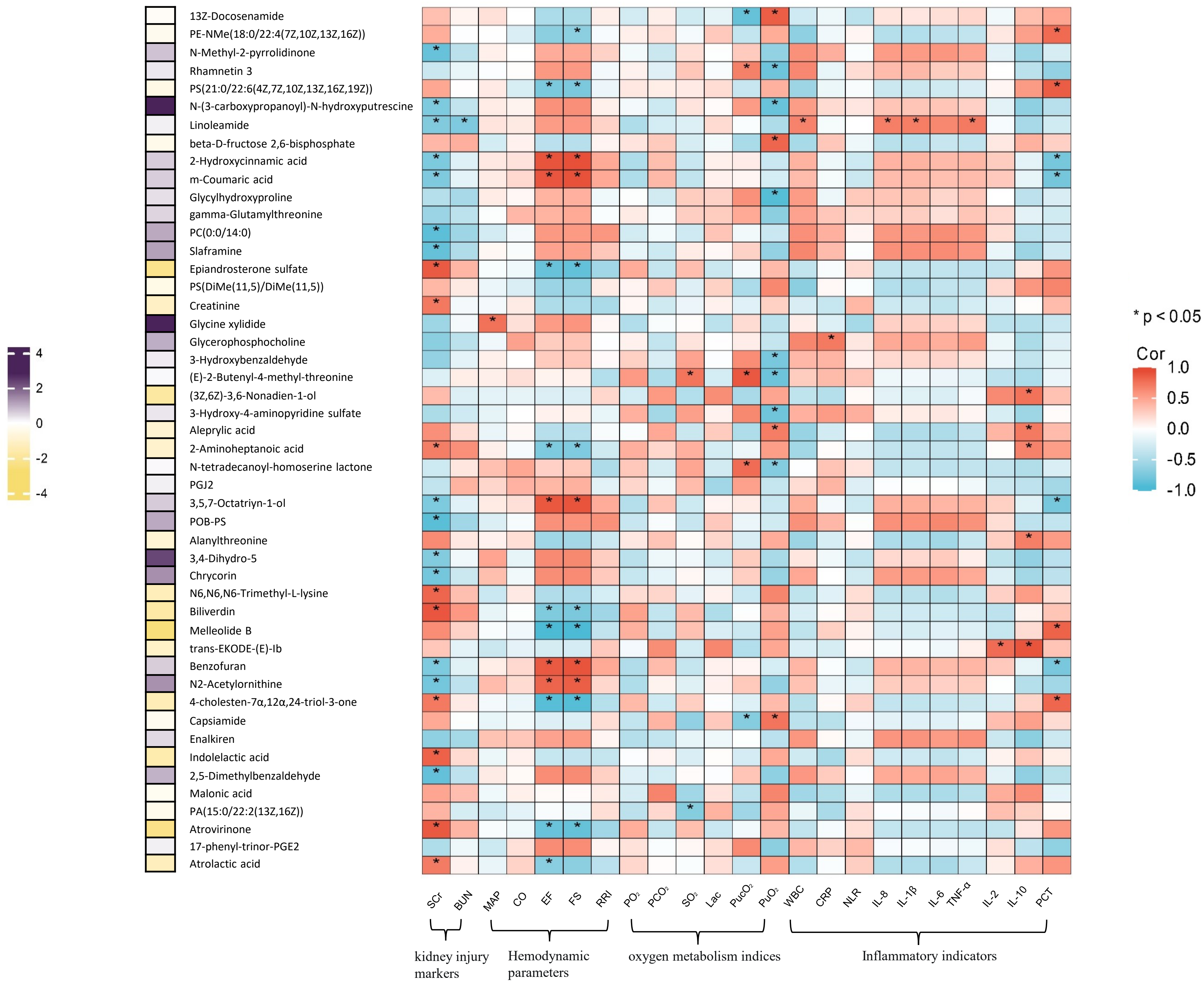
13Z-Docosenamide
PE-NMe(18:0/22:4(7Z,10Z,13Z,16Z))
N-Methyl-2-pyrrolidinone
Rhamnetin 3
PS(21:0/22:6(4Z,7Z,10Z,13Z,16Z,19Z))
N-(3-carboxypropanoyl)-N-hydroxyputrescine
Linoleamide
beta-D-fructose 2,6-bisphosphate
2-Hydroxycinnamic acid
m-Coumaric acid
Glycylhydroxyproline
gamma-Glutamylthreonine
PC(0:0/14:0)
Slaframine
Epiandrosterone sulfate
PS(DiMe(11,5)/DiMe(11,5))
Creatinine
Glycine xylidide
Glycerophosphocholine
3-Hydroxybenzaldehyde
(E)-2-Butenyl-4-methyl-threonine
(3Z,6Z)-3,6-Nonadien-1-ol
3-Hydroxy-4-aminopyridine sulfate
Aleprylic acid
2-Aminoheptanoic acid
N-tetradecanoyl-homoserine lactone
PGJ2
3,5,7-Octatriyn-1-ol
POB-PS
Alanylthreonine
3,4-Dihydro-5
Chrycorin
N6,N6,N6-Trimethyl-L-lysine
Biliverdin
Melleolide B
trans-EKODE-(E)-Ib
Benzofuran
N2-Acetylornithine
4-cholesten-7α,12α,24-triol-3-one
Capsiamide
Enalkiren
Indolelactic acid
2,5-Dimethylbenzaldehyde
Malonic acid
PA(15:0/22:2(13Z,16Z))
Atrovirinone
17-phenyl-trinor-PGE2
Atrolactic acid
4
2
0
-2
-4
SCr
BUN
MAP
CO
EF
FS
RRI
PO2
PCO2
SO2
Lac
PucO2
PuO2
WBC
CRP
NLR
IL-8
IL-1β
IL-6
TNF-α
IL-2
IL-10
PCT
kidney injury markers
Hemodynamic parameters
oxygen metabolism indices
Inflammatory indicators
* p < 0.05
Cor
1.0
0.5
0.0
-0.5
-1.0

Figure S2 C

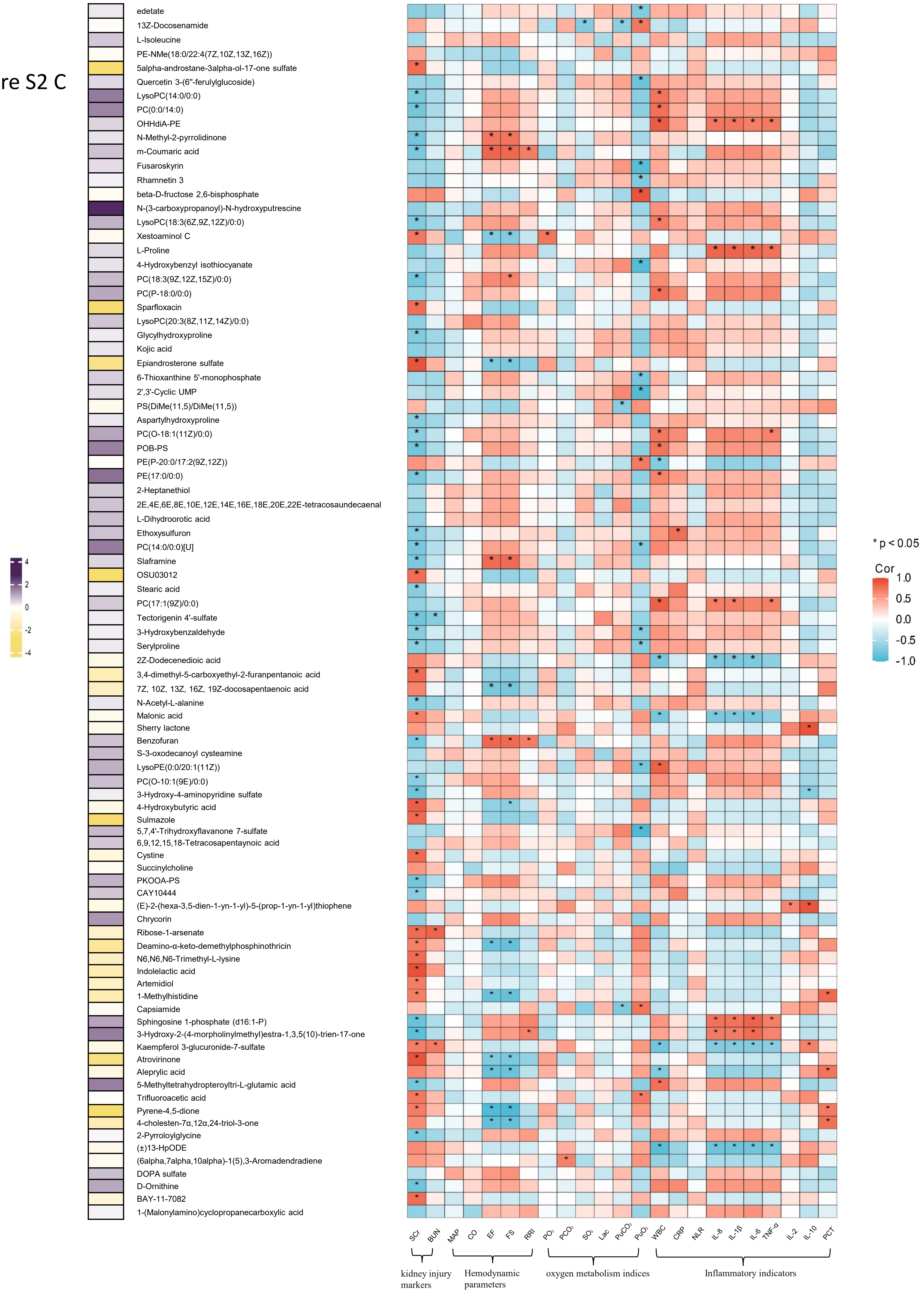
edetate
13Z-Docosenamide
L-Isoleucine
PE-NMe(18:0/22:4(7Z,10Z,13Z,16Z))
5alpha-androstane-3alpha-ol-17-one sulfate
Quercetin 3-(6''-ferulylglucoside)
LysoPC(14:0/0:0)
PC(0:0/14:0)
OHHdiA-PE
N-Methyl-2-pyrrolidinone
m-Coumaric acid
Fusaroskyrin
Rhamnetin 3
beta-D-fructose 2,6-bisphosphate
N-(3-carboxypropanoyl)-N-hydroxyputrescine
LysoPC(18:3(6Z,9Z,12Z)/0:0)
Xestoaminol C
L-Proline
4-Hydroxybenzyl isothiocyanate
PC(18:3(9Z,12Z,15Z)/0:0)
PC(P-18:0/0:0)
Sparfloxacin
LysoPC(20:3(8Z,11Z,14Z)/0:0)
Glycylhydroxyproline
Kojic acid
Epiandrosterone sulfate
6-Thioxanthine 5'-monophosphate
2',3'-Cyclic UMP
PS(DiMe(11,5)/DiMe(11,5))
Aspartylhydroxyproline
PC(O-18:1(11Z)/0:0)
POB-PS
PE(P-20:0/17:2(9Z,12Z))
PE(17:0/0:0)
2-Heptanethiol
2E,4E,6E,8E,10E,12E,14E,16E,18E,20E,22E-tetracosaundecaenal
L-Dihydroorotic acid
Ethoxysulfuron
PC(14:0/0:0)[U]
Slaframine
OSU03012
Stearic acid
PC(17:1(9Z)/0:0)
Tectorigenin 4'-sulfate
3-Hydroxybenzaldehyde
Serylproline
2Z-Dodecenedioic acid
3,4-dimethyl-5-carboxyethyl-2-furanpentanoic acid
7Z, 10Z, 13Z, 16Z, 19Z-docosapentaenoic acid
N-Acetyl-L-alanine
Malonic acid
Sherry lactone
Benzofuran
S-3-oxodecanoyl cysteamine
LysoPE(0:0/20:1(11Z))
PC(O-10:1(9E)/0:0)
3-Hydroxy-4-aminopyridine sulfate
4-Hydroxybutyric acid
Sulmazole
5,7,4'-Trihydroxyflavanone 7-sulfate
6,9,12,15,18-Tetracosapentaynoic acid
Cystine
Succinylcholine
PKOOA-PS
CAY10444
(E)-2-(hexa-3,5-dien-1-yn-1-yl)-5-(prop-1-yn-1-yl)thiophene
Chrycorin
Ribose-1-arsenate
Deamino-α-keto-demethylphosphinothricin
N6,N6,N6-Trimethyl-L-lysine
Indolelactic acid
Artemidiol
1-Methylhistidine
Capsiamide
Sphingosine 1-phosphate (d16:1-P)
3-Hydroxy-2-(4-morpholinylmethyl)estra-1,3,5(10)-trien-17-one
Kaempferol 3-glucuronide-7-sulfate
Atrovirinone
Aleprylic acid
5-Methyltetrahydropteroyltri-L-glutamic acid
Trifluoroacetic acid
Pyrene-4,5-dione
4-cholesten-7α,12α,24-triol-3-one
2-Pyrroloylglycine
(±)13-HpODE
(6alpha,7alpha,10alpha)-1(5),3-Aromadendradiene
DOPA sulfate
D-Ornithine
BAY-11-7082
1-(Malonylamino)cyclopropanecarboxylic acid
4
2
0
-2
-4
SCr
BUN
MAP
CO
EF
FS
RRI
PO2
PCO2
SO2
Lac
PuCO2
PuO2
WBC
CRP
NLR
IL-8
IL-1β
IL-6
TNF-α
IL-2
IL-10
PCT
kidney injury markers
Hemodynamic parameters
oxygen metabolism indices
Inflammatory indicators
* p < 0.05
Cor
1.0
0.5
0.0
-0.5
-1.0

Figure S2 The left heat map illustrates the log2fold change (FC) of the serum metabolites' expression levels. The color scale ranges from purple, indicating high expression, to yellow, indicating low expression. The closer the color is to bright purple, the higher the expression in the Non-AKI group; conversely, the closer it is to bright yellow, the higher the expression in the SA-AKI group. The right heat map displays the Pearson's correlation between the serum metabolites and clinical parameters at three different time points: D0 (A), D1 (B), and D2 (C). If the coefficient in the interaction term is greater than 0 (red), it suggests that the serum metabolites' expression levels increase in the presence of the factor. On the other hand, a coefficient less than 0 (blue) implies that the serum metabolites' expression levels decrease in the presence of the factor.
AKI=acute kidney injury, SCr=serum creatinine, BUN=blood urea nitrogen, MAP=mean arterial pressure, CO=cardiac output, FS=fractional shortening., EF=ejection fraction, RRI=renal resistive index, PaO_2=pressure of oxygen in artery, $PaCO_2$=partial pressure of carbon dioxide in artery, SaO_2=saturation of arterial blood oxygen, Lac=lactic acid, PuO_2=pressure of oxygen in urine, $PuCO_2$=partial pressure of carbon dioxide in urine, WBC=white blood cell, NLR=the neutrophil to lymphocyte ratio, PCT=procalcitonin, Corr=correlation.
