# Supplementary material for: Investigating gut microbiota–blood and urine metabolite correlations in early sepsis-induced acute kidney injury: insights from targeted KEGG analyses
Source: Front Cell Infect Microbiol. 2024 Jun 3;14:1375874. doi: 10.3389/fcimb.2024.1375874 (PMC11180806; doi:10.3389/fcimb.2024.1375874)
Supplement: Supplementary file 3 [file DataSheet_3.pdf]

Figure S3 A

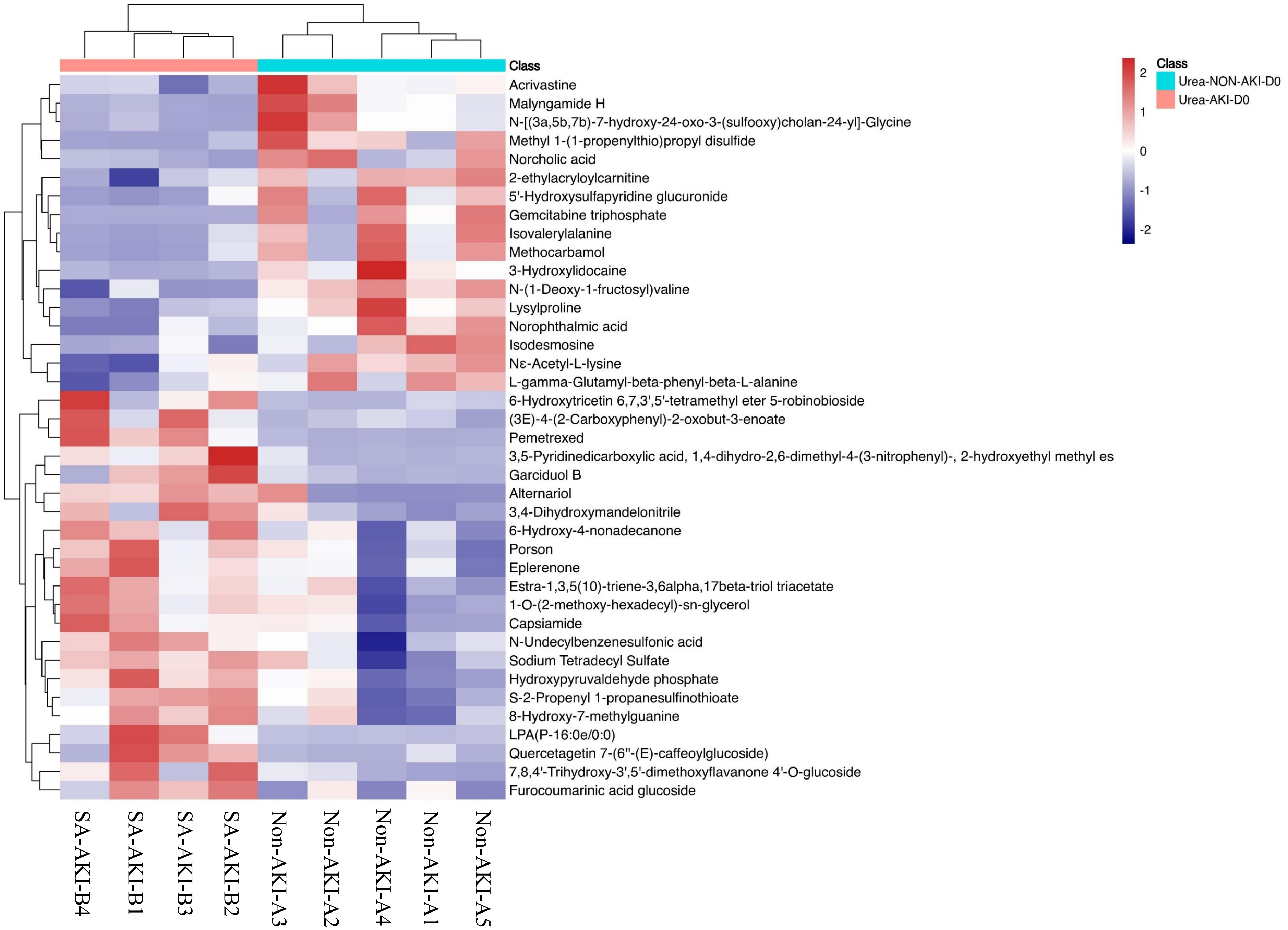
Class
Acrivastine
Malyngamide H
N-[(3a,5b,7b)-7-hydroxy-24-oxo-3-(sulfooxy)cholan-24-yl]-Glycine
Methyl 1-(1-propenylthio)propyl disulfide
Norcholic acid
2-ethylacryloylcarnitine
5'-Hydroxysulfapyridine glucuronide
Gemcitabine triphosphate
Isovalerylalanine
Methocarbamol
3-Hydroxylidocaine
N-(1-Deoxy-1-fructosyl)valine
Lysylproline
Norophthalmic acid
Isodesmosine
Nε-Acetyl-L-lysine
L-gamma-Glutamyl-beta-phenyl-beta-L-alanine
6-Hydroxytricetin 6,7,3',5'-tetramethyl eter 5-robinobioside
(3E)-4-(2-Carboxyphenyl)-2-oxobut-3-enoate
Pemetrexed
3,5-Pyridinedicarboxylic acid, 1,4-dihydro-2,6-dimethyl-4-(3-nitrophenyl)-, 2-hydroxyethyl methyl es
Garciduol B
Alternariol
3,4-Dihydroxymandelonitrile
6-Hydroxy-4-nonadecanone
Porson
Eplerenone
Estra-1,3,5(10)-triene-3,6alpha,17beta-triol triacetate
1-O-(2-methoxy-hexadecyl)-sn-glycerol
Capsiamide
N-Undecylbenzenesulfonic acid
Sodium Tetradecyl Sulfate
Hydroxypyruvaldehyde phosphate
S-2-Propenyl 1-propanesulfinothioate
8-Hydroxy-7-methylguanine
LPA(P-16:0e/0:0)
Quercetagetin 7-(6''-(E)-caffeoylglucoside)
7,8,4'-Trihydroxy-3',5'-dimethoxyflavanone 4'-O-glucoside
Furocoumarinic acid glucoside
SA-AKI-B4
SA-AKI-B1
SA-AKI-B3
SA-AKI-B2
Non-AKI-A3
Non-AKI-A2
Non-AKI-A4
Non-AKI-A1
Non-AKI-A5
2
1
0
-1
-2
Class
Urea-NON-AKI-D0
Urea-AKI-D0

Figure S3 B

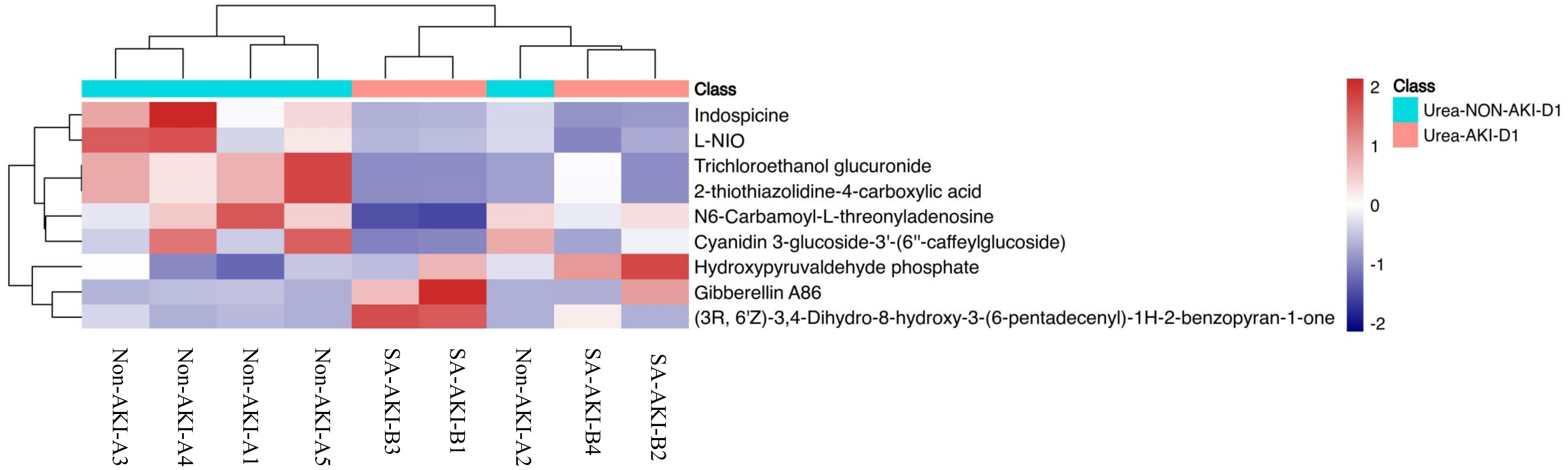

Class
Indospicine
L-NIO
Trichloroethanol glucuronide
2-thiothiazolidine-4-carboxylic acid
N6-Carbamoyl-L-threonyladenosine
Cyanidin 3-glucoside-3'-(6''-caffeylglucoside)
Hydroxypyruvaldehyde phosphate
Gibberellin A86
(3R, 6'Z)-3,4-Dihydro-8-hydroxy-3-(6-pentadecenyl)-1H-2-benzopyran-1-one
Non-AKI-A3
Non-AKI-A4
Non-AKI-A1
Non-AKI-A5
SA-AKI-B3
SA-AKI-B1
Non-AKI-A2
SA-AKI-B4
SA-AKI-B2
2
1
0
-1
-2
Class
Urea-NON-AKI-D1
Urea-AKI-D1

Figure S3 C

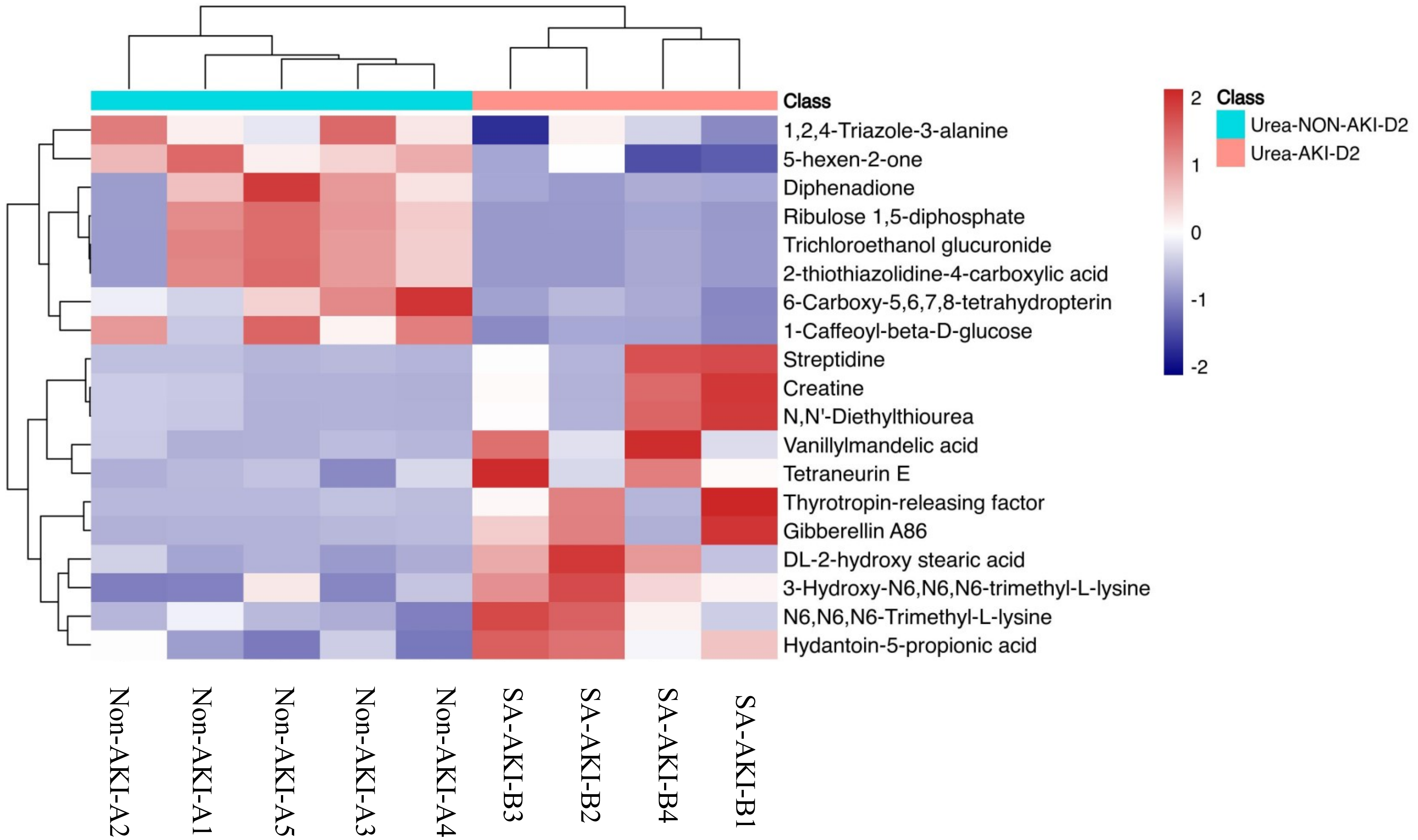

Figure S3. Heatmap displaying the hierarchical clustering of the urinary metabolite enrichment analysis at D0 (A), D1 (B), and D2 (C).
