# Supplementary material for: Investigating gut microbiota–blood and urine metabolite correlations in early sepsis-induced acute kidney injury: insights from targeted KEGG analyses
Source: Front Cell Infect Microbiol. 2024 Jun 3;14:1375874. doi: 10.3389/fcimb.2024.1375874 (PMC11180806; doi:10.3389/fcimb.2024.1375874)
Supplement: Supplementary file 4 [file DataSheet_4.pdf]

Figure S4A(1)

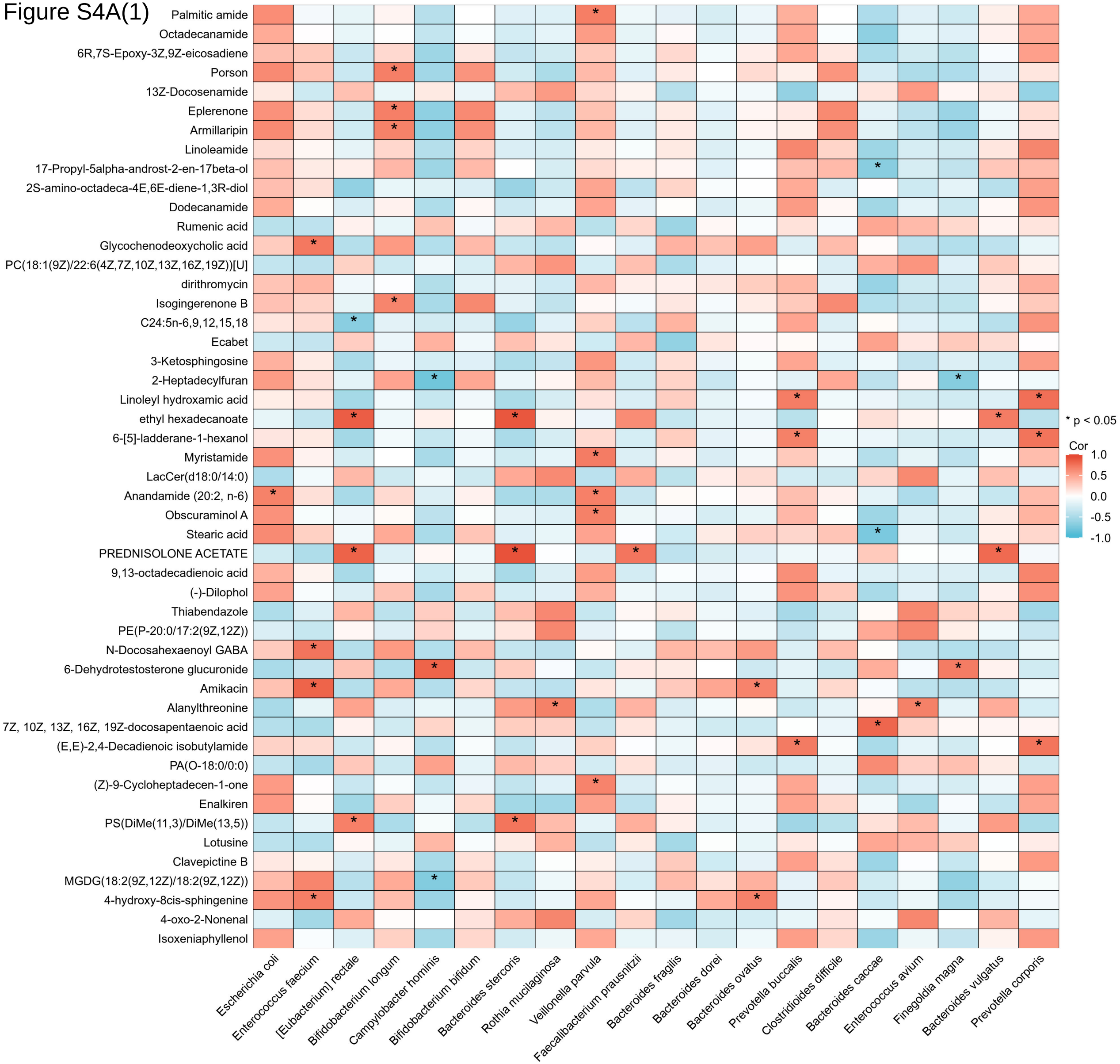
Palmitic amide
Octadecanamide
6R,7S-Epoxy-3Z,9Z-eicosadiene
Porson
13Z-Docosenamide
Eplerenone
Armillaripin
Linoleamide
17-Propyl-5alpha-androst-2-en-17beta-ol
2S-amino-octadeca-4E,6E-diene-1,3R-diol
Dodecanamide
Rumenic acid
Glycochenodeoxycholic acid
PC(18:1(9Z)/22:6(4Z,7Z,10Z,13Z,16Z,19Z))[U]
dirithromycin
Isogingerenone B
C24:5n-6,9,12,15,18
Ecabet
3-Ketosphingosine
2-Heptadecylfuran
Linoleyl hydroxamic acid
ethyl hexadecanoate
6-[5]-ladderane-1-hexanol
Myristamide
LacCer(d18:0/14:0)
Anandamide (20:2, n-6)
Obscuraminol A
Stearic acid
PREDNISOLONE ACETATE
9,13-octadecadienoic acid
(-)-Dilophol
Thiabendazole
PE(P-20:0/17:2(9Z,12Z))
N-Docosahexaenoyl GABA
6-Dehydrotestosterone glucuronide
Amikacin
Alanylthreonine
7Z, 10Z, 13Z, 16Z, 19Z-docosapentaenoic acid
(E,E)-2,4-Decadienoic isobutylamide
PA(O-18:0/0:0)
(Z)-9-Cycloheptadecen-1-one
Enalkiren
PS(DiMe(11,3)/DiMe(13,5))
Lotusine
Clavepictine B
MGDG(18:2(9Z,12Z)/18:2(9Z,12Z))
4-hydroxy-8cis-sphingenine
4-oxo-2-Nonenal
Isoxeniaphyllenol
Escherichia coli
Enterococcus faecium
[Eubacterium] rectale
Bifidobacterium longum
Campylobacter hominis
Bifidobacterium bifidum
Bacteroides stercoris
Rothia mucilaginosa
Veillonella parvula
Faecalibacterium prausnitzii
Bacteroides fragilis
Bacteroides dorei
Bacteroides ovatus
Prevotella buccalis
Clostridioides difficile
Bacteroides caccae
Enterococcus avium
Finegoldia magna
Bacteroides vulgatus
Prevotella corporis
* p < 0.05
Cor
1.0
0.5
0.0
-0.5
-1.0

Figure S4 A(2)

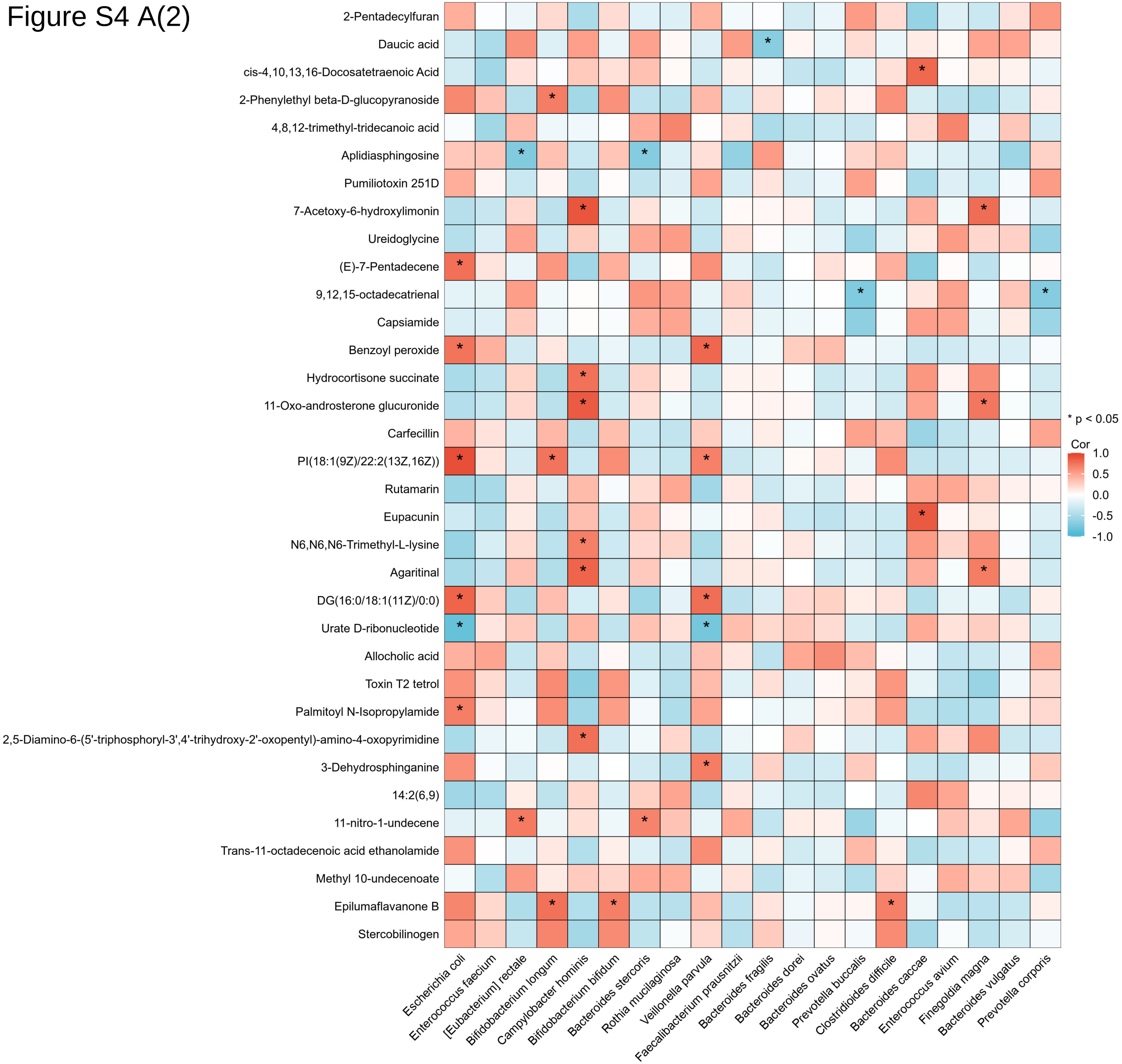
2-Pentadecylfuran
Daucic acid
cis-4,10,13,16-Docosatetraenoic Acid
2-Phenylethyl beta-D-glucopyranoside
4,8,12-trimethyl-tridecanoic acid
Aplidiasphingosine
Pumiliotoxin 251D
7-Acetoxy-6-hydroxylimonin
Ureidoglycine
(E)-7-Pentadecene
9,12,15-octadecatrienal
Capsiamide
Benzoyl peroxide
Hydrocortisone succinate
11-Oxo-androsterone glucuronide
Carfecillin
PI(18:1(9Z)/22:2(13Z,16Z))
Rutamarin
Eupacunin
N6,N6,N6-Trimethyl-L-lysine
Agaritinal
DG(16:0/18:1(11Z)/0:0)
Urate D-ribonucleotide
Allocholic acid
Toxin T2 tetrol
Palmitoyl N-Isopropylamide
2,5-Diamino-6-(5'-triphosphoryl-3',4'-trihydroxy-2'-oxopentyl)-amino-4-oxopyrimidine
3-Dehydrosphinganine
14:2(6,9)
11-nitro-1-undecene
Trans-11-octadecenoic acid ethanolamide
Methyl 10-undecenoate
Epilumaflavanone B
Stercobilinogen
Escherichia coli
Enterococcus faecium
[Eubacterium] rectale
Bifidobacterium longum
Campylobacter hominis
Bifidobacterium bifidum
Bacteroides stercoris
Rothia mucilaginosa
Veillonella parvula
Faecalibacterium prausnitzii
Bacteroides fragilis
Bacteroides dorei
Bacteroides ovatus
Prevotella buccalis
Clostridioides difficile
Bacteroides caccae
Enterococcus avium
Finegoldia magna
Bacteroides vulgatus
Prevotella corporis
* p < 0.05
Cor
1.0
0.5
0.0
-0.5
-1.0

Figure S4 B

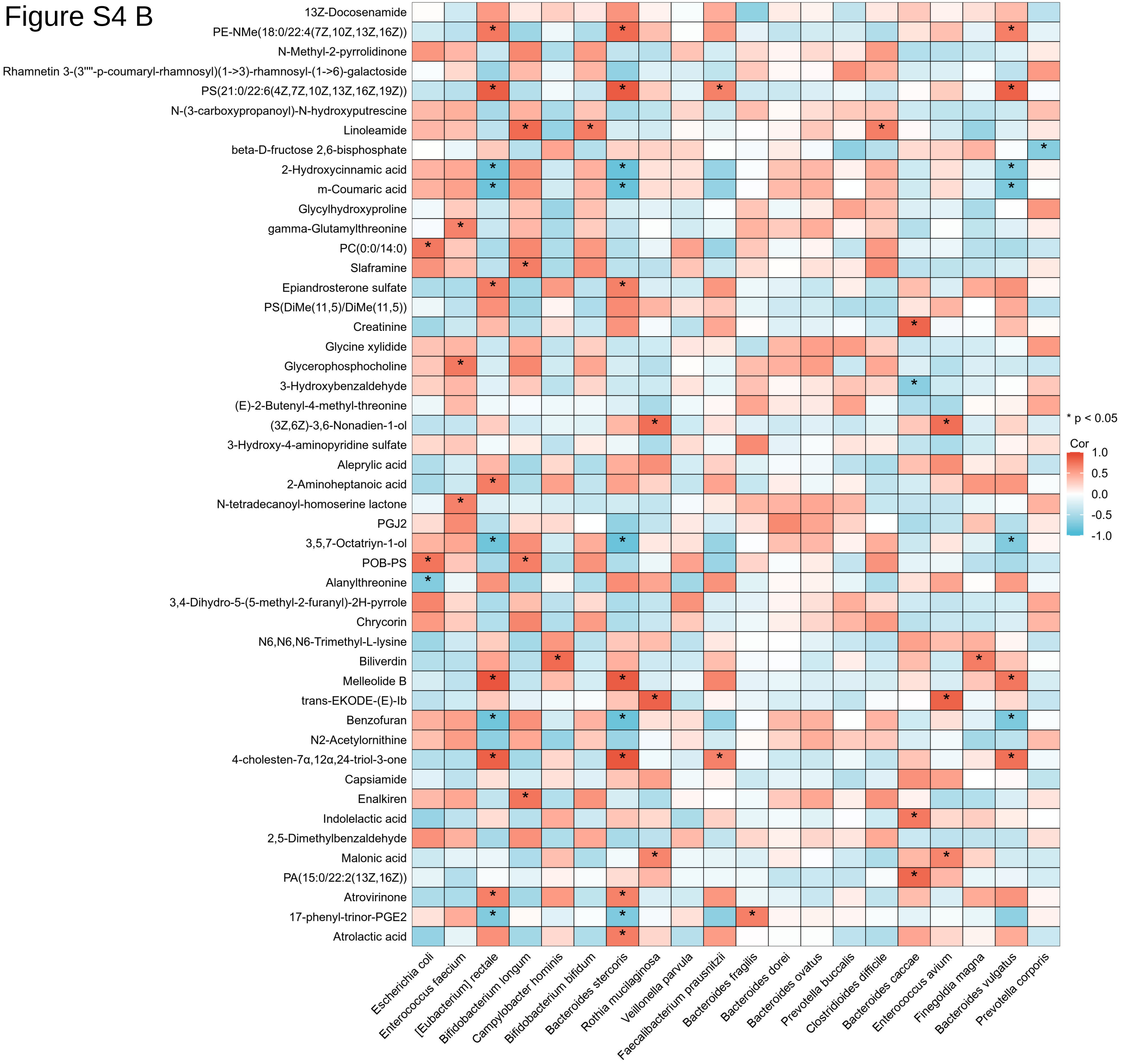
13Z-Docosenamide
PE-NMe(18:0/22:4(7Z,10Z,13Z,16Z))
N-Methyl-2-pyrrolidinone
Rhamnetin 3-(3''''-p-coumaryl-rhamnosyl)(1->3)-rhamnosyl-(1->6)-galactoside
PS(21:0/22:6(4Z,7Z,10Z,13Z,16Z,19Z))
N-(3-carboxypropanoyl)-N-hydroxyputrescine
Linoleamide
beta-D-fructose 2,6-bisphosphate
2-Hydroxycinnamic acid
m-Coumaric acid
Glycylhydroxyproline
gamma-Glutamylthreonine
PC(0:0/14:0)
Slaframine
Epiandrosterone sulfate
PS(DiMe(11,5)/DiMe(11,5))
Creatinine
Glycine xylidide
Glycerophosphocholine
3-Hydroxybenzaldehyde
(E)-2-Butenyl-4-methyl-threonine
(3Z,6Z)-3,6-Nonadien-1-ol
3-Hydroxy-4-aminopyridine sulfate
Aleprylic acid
2-Aminoheptanoic acid
N-tetradecanoyl-homoserine lactone
PGJ2
3,5,7-Octatriyn-1-ol
POB-PS
Alanylthreonine
3,4-Dihydro-5-(5-methyl-2-furanyl)-2H-pyrrole
Chrycorin
N6,N6,N6-Trimethyl-L-lysine
Biliverdin
Melleolide B
trans-EKODE-(E)-Ib
Benzofuran
N2-Acetylornithine
4-cholesten-7α,12α,24-triol-3-one
Capsiamide
Enalkiren
Indolelactic acid
2,5-Dimethylbenzaldehyde
Malonic acid
PA(15:0/22:2(13Z,16Z))
Atrovirinone
17-phenyl-trinor-PGE2
Atrolactic acid
Escherichia coli
Enterococcus faecium
[Eubacterium] rectale
Bifidobacterium longum
Campylobacter hominis
Bifidobacterium bifidum
Bacteroides stercoris
Rothia mucilaginosa
Veillonella parvula
Faecalibacterium prausnitzii
Bacteroides fragilis
Bacteroides dorei
Bacteroides ovatus
Prevotella buccalis
Clostridioides difficile
Bacteroides caccae
Enterococcus avium
Finegoldia magna
Bacteroides vulgatus
Prevotella corporis
* p < 0.05
Cor
1.0
0.5
0.0
-0.5
-1.0

Figure S4 C(1)

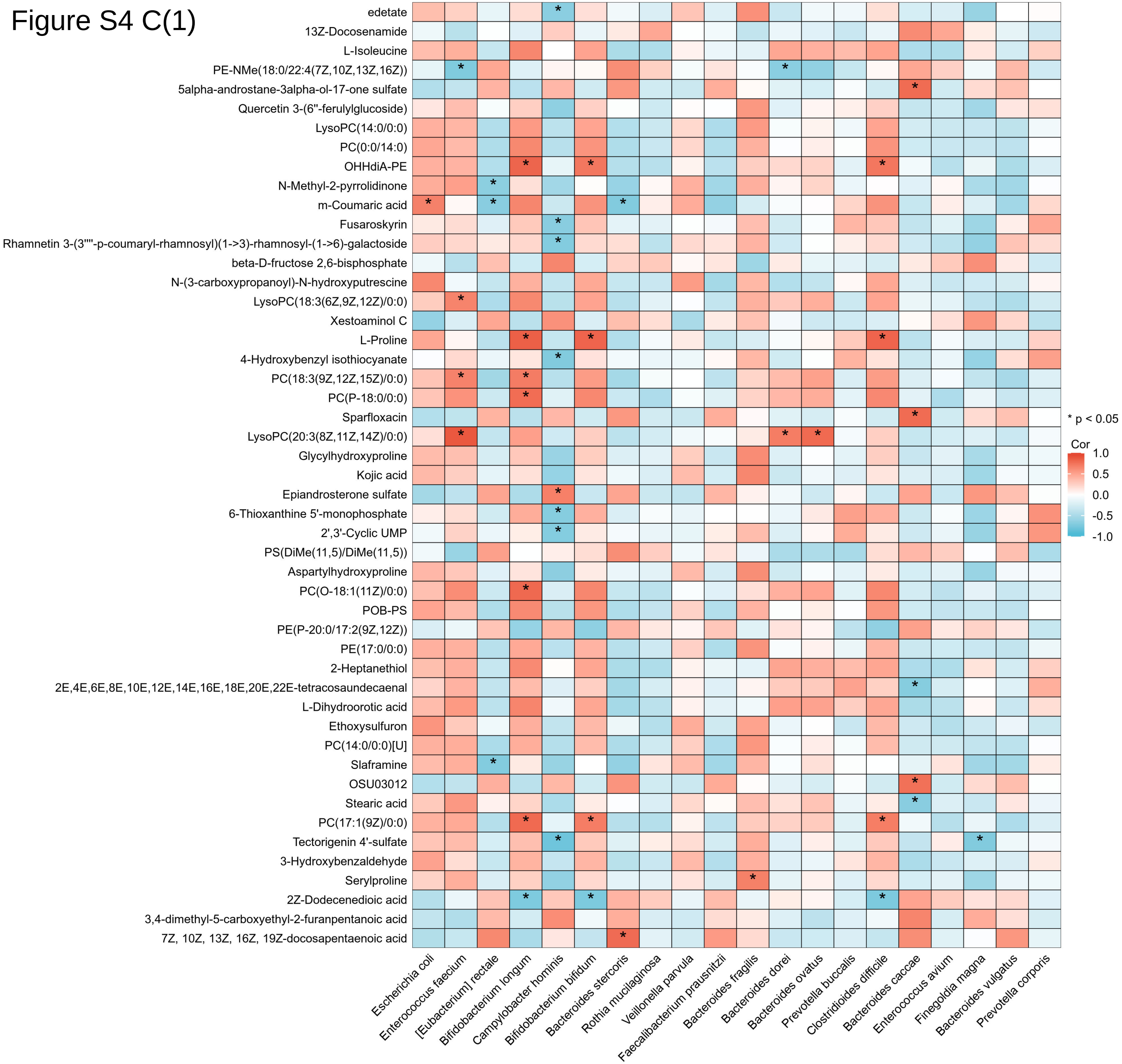
edetate
13Z-Docosenamide
L-Isoleucine
PE-NMe(18:0/22:4(7Z,10Z,13Z,16Z))
5alpha-androstane-3alpha-ol-17-one sulfate
Quercetin 3-(6''-ferulylglucoside)
LysoPC(14:0/0:0)
PC(0:0/14:0)
OHHdiA-PE
N-Methyl-2-pyrrolidinone
m-Coumaric acid
Fusaroskyrin
Rhamnetin 3-(3''''-p-coumaryl-rhamnosyl)(1->3)-rhamnosyl-(1->6)-galactoside
beta-D-fructose 2,6-bisphosphate
N-(3-carboxypropanoyl)-N-hydroxyputrescine
LysoPC(18:3(6Z,9Z,12Z)/0:0)
Xestoaminol C
L-Proline
4-Hydroxybenzyl isothiocyanate
PC(18:3(9Z,12Z,15Z)/0:0)
PC(P-18:0/0:0)
Sparfloxacin
LysoPC(20:3(8Z,11Z,14Z)/0:0)
Glycylhydroxyproline
Kojic acid
Epiandrosterone sulfate
6-Thioxanthine 5'-monophosphate
2',3'-Cyclic UMP
PS(DiMe(11,5)/DiMe(11,5))
Aspartylhydroxyproline
PC(O-18:1(11Z)/0:0)
POB-PS
PE(P-20:0/17:2(9Z,12Z))
PE(17:0/0:0)
2-Heptanethiol
2E,4E,6E,8E,10E,12E,14E,16E,18E,20E,22E-tetracosaundecaenal
L-Dihydroorotic acid
Ethoxysulfuron
PC(14:0/0:0)[U]
Slaframine
OSU03012
Stearic acid
PC(17:1(9Z)/0:0)
Tectorigenin 4'-sulfate
3-Hydroxybenzaldehyde
Serylproline
2Z-Dodecenedioic acid
3,4-dimethyl-5-carboxyethyl-2-furanpentanoic acid
7Z, 10Z, 13Z, 16Z, 19Z-docosapentaenoic acid
Escherichia coli
Enterococcus faecium
[Eubacterium] rectale
Bifidobacterium longum
Campylobacter hominis
Bifidobacterium bifidum
Bacteroides stercoris
Rothia mucilaginosa
Veillonella parvula
Faecalibacterium prausnitzii
Bacteroides fragilis
Bacteroides dorei
Bacteroides ovatus
Prevotella buccalis
Clostridioides difficile
Bacteroides caccae
Enterococcus avium
Finegoldia magna
Bacteroides vulgatus
Prevotella corporis
* p < 0.05
Cor
1.0
0.5
0.0
-0.5
-1.0

Figure S4 C(2)

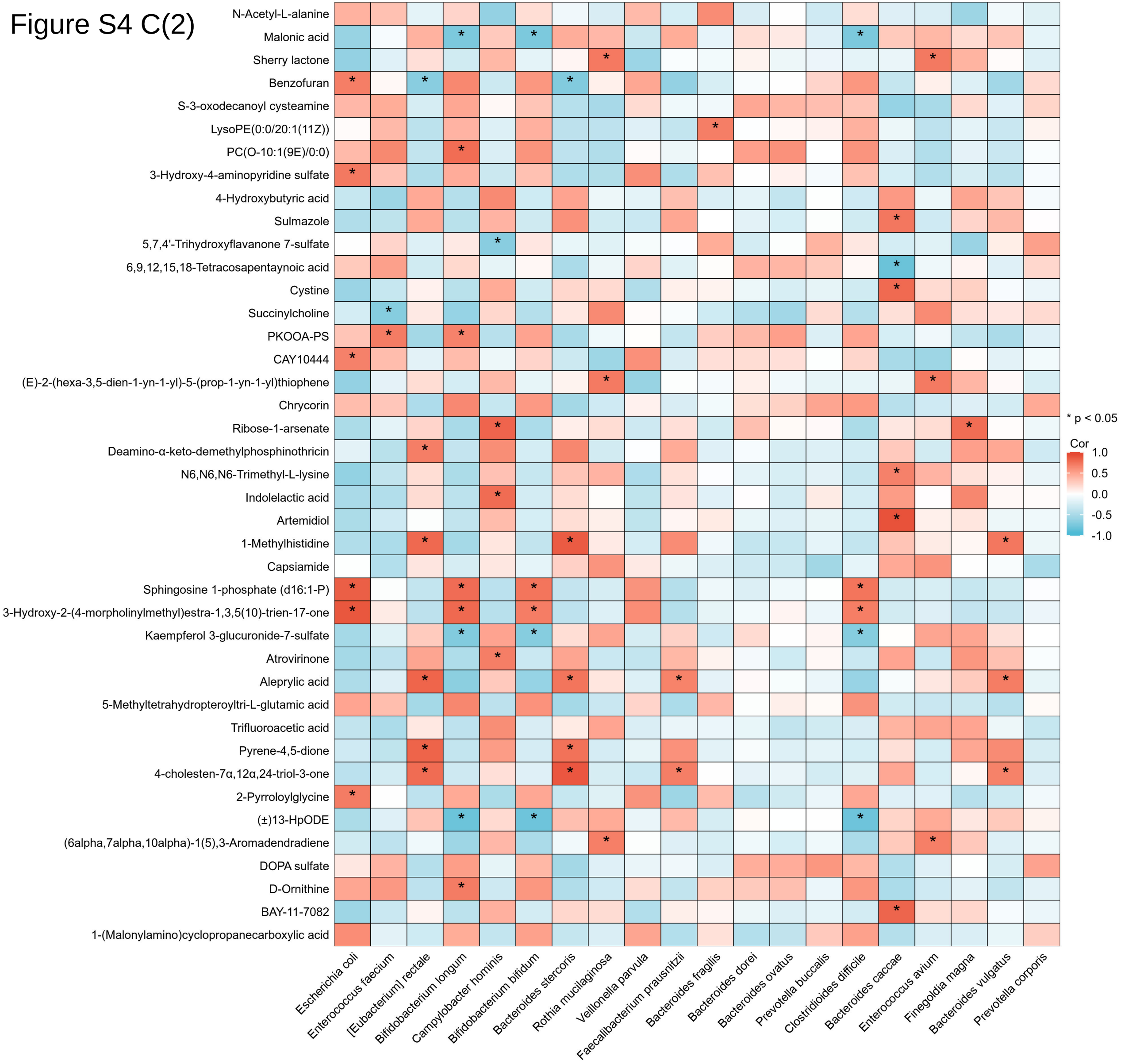
N-Acetyl-L-alanine
Malonic acid
Sherry lactone
Benzofuran
S-3-oxodecanoyl cysteamine
LysoPE(0:0/20:1(11Z))
PC(O-10:1(9E)/0:0)
3-Hydroxy-4-aminopyridine sulfate
4-Hydroxybutyric acid
Sulmazole
5,7,4'-Trihydroxyflavanone 7-sulfate
6,9,12,15,18-Tetracosapentaynoic acid
Cystine
Succinylcholine
PKOOA-PS
CAY10444
(E)-2-(hexa-3,5-dien-1-yn-1-yl)-5-(prop-1-yn-1-yl)thiophene
Chrycorin
Ribose-1-arsenate
Deamino-α-keto-demethylphosphinothricin
N6,N6,N6-Trimethyl-L-lysine
Indolelactic acid
Artemidiol
1-Methylhistidine
Capsiamide
Sphingosine 1-phosphate (d16:1-P)
3-Hydroxy-2-(4-morpholinylmethyl)estra-1,3,5(10)-trien-17-one
Kaempferol 3-glucuronide-7-sulfate
Atrovirinone
Aleprylic acid
5-Methyltetrahydropteroyltri-L-glutamic acid
Trifluoroacetic acid
Pyrene-4,5-dione
4-cholesten-7α,12α,24-triol-3-one
2-Pyrroloylglycine
(±)13-HpODE
(6alpha,7alpha,10alpha)-1(5),3-Aromadendradiene
DOPA sulfate
D-Ornithine
BAY-11-7082
1-(Malonylamino)cyclopropanecarboxylic acid
Escherichia coli
Enterococcus faecium
[Eubacterium] rectale
Bifidobacterium longum
Campylobacter hominis
Bifidobacterium bifidum
Bacteroides stercoris
Rothia mucilaginosa
Veillonella parvula
Faecalibacterium prausnitzii
Bacteroides fragilis
Bacteroides dorei
Bacteroides ovatus
Prevotella buccalis
Clostridioides difficile
Bacteroides caccae
Enterococcus avium
Finegoldia magna
Bacteroides vulgatus
Prevotella corporis
* p < 0.05
Cor
1.0
0.5
0.0
-0.5
-1.0

Figure S4 (A) shows a Pearson's correlation heat map depicting the relationship between the gut microbiota at D0 and blood metabolites at D0 (A), D1 (B), and D2 (C). A positive coefficient for the interaction term (highlighted in red) indicates higher expression levels of the gut microbiota in association with the specified factor. Conversely, a negative coefficient (displayed in blue) denotes a decrease in the gut microbiota's expression levels when the factor is present.
