# Supplementary material for: Investigating gut microbiota–blood and urine metabolite correlations in early sepsis-induced acute kidney injury: insights from targeted KEGG analyses
Source: Front Cell Infect Microbiol. 2024 Jun 3;14:1375874. doi: 10.3389/fcimb.2024.1375874 (PMC11180806; doi:10.3389/fcimb.2024.1375874)
Supplement: Supplementary file 5 [file DataSheet_5.pdf]

Figure S5 A

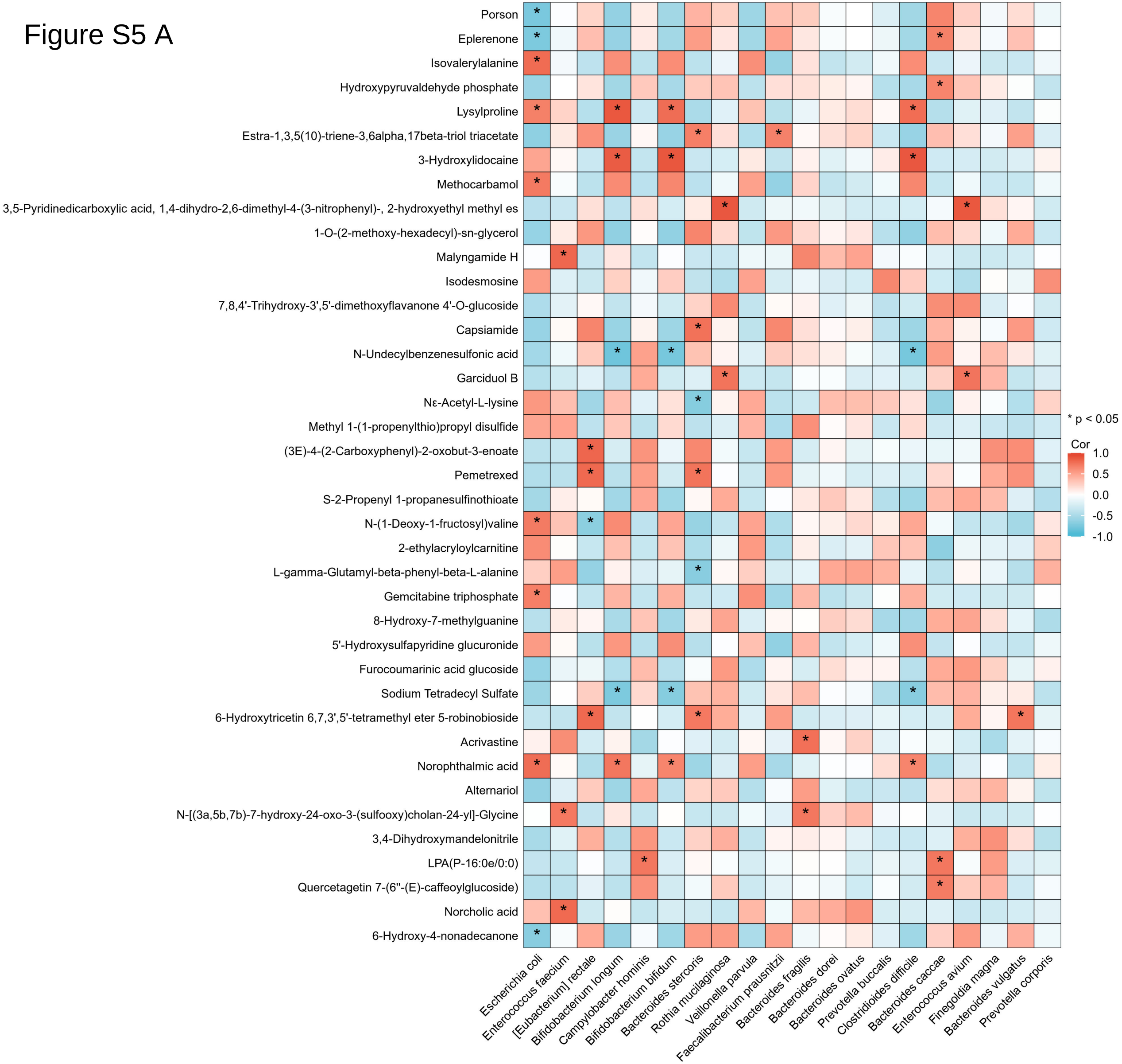

Porson
Eplerenone
Isovalerylalanine
Hydroxypyruvaldehyde phosphate
Lysylproline
Estra-1,3,5(10)-triene-3,6alpha,17beta-triol triacetate
3-Hydroxylidocaine
Methocarbamol
3,5-Pyridinedicarboxylic acid, 1,4-dihydro-2,6-dimethyl-4-(3-nitrophenyl)-, 2-hydroxyethyl methyl es
1-O-(2-methoxy-hexadecyl)-sn-glycerol
Malyngamide H
Isodesmosine
7,8,4'-Trihydroxy-3',5'-dimethoxyflavanone 4'-O-glucoside
Capsiamide
N-Undecylbenzenesulfonic acid
Garciduol B
Nε-Acetyl-L-lysine
Methyl 1-(1-propenylthio)propyl disulfide
(3E)-4-(2-Carboxyphenyl)-2-oxobut-3-enoate
Pemetrexed
S-2-Propenyl 1-propanesulfinothioate
N-(1-Deoxy-1-fructosyl)valine
2-ethylacryloylcarnitine
L-gamma-Glutamyl-beta-phenyl-beta-L-alanine
Gemcitabine triphosphate
8-Hydroxy-7-methylguanine
5'-Hydroxysulfapyridine glucuronide
Furocoumarinic acid glucoside
Sodium Tetradecyl Sulfate
6-Hydroxytricetin 6,7,3',5'-tetramethyl eter 5-robinobioside
Acrivastine
Norophthalmic acid
Alternariol
N-[(3a,5b,7b)-7-hydroxy-24-oxo-3-(sulfooxy)cholan-24-yl]-Glycine
3,4-Dihydroxymandelonitrile
LPA(P-16:0e/0:0)
Quercetagetin 7-(6''-(E)-caffeoylglucoside)
Norcholic acid
6-Hydroxy-4-nonadecanone
Escherichia coli
Enterococcus faecium
[Eubacterium] rectale
Bifidobacterium longum
Campylobacter hominis
Bifidobacterium bifidum
Bacteroides stercoris
Rothia mucilaginosa
Veillonella parvula
Faecalibacterium prausnitzii
Bacteroides fragilis
Bacteroides dorei
Bacteroides ovatus
Prevotella buccalis
Clostridioides difficile
Bacteroides caccae
Enterococcus avium
Finegoldia magna
Bacteroides vulgatus
Prevotella corporis
* p < 0.05
Cor
1.0
0.5
0.0
-0.5
-1.0

Figure S5 B

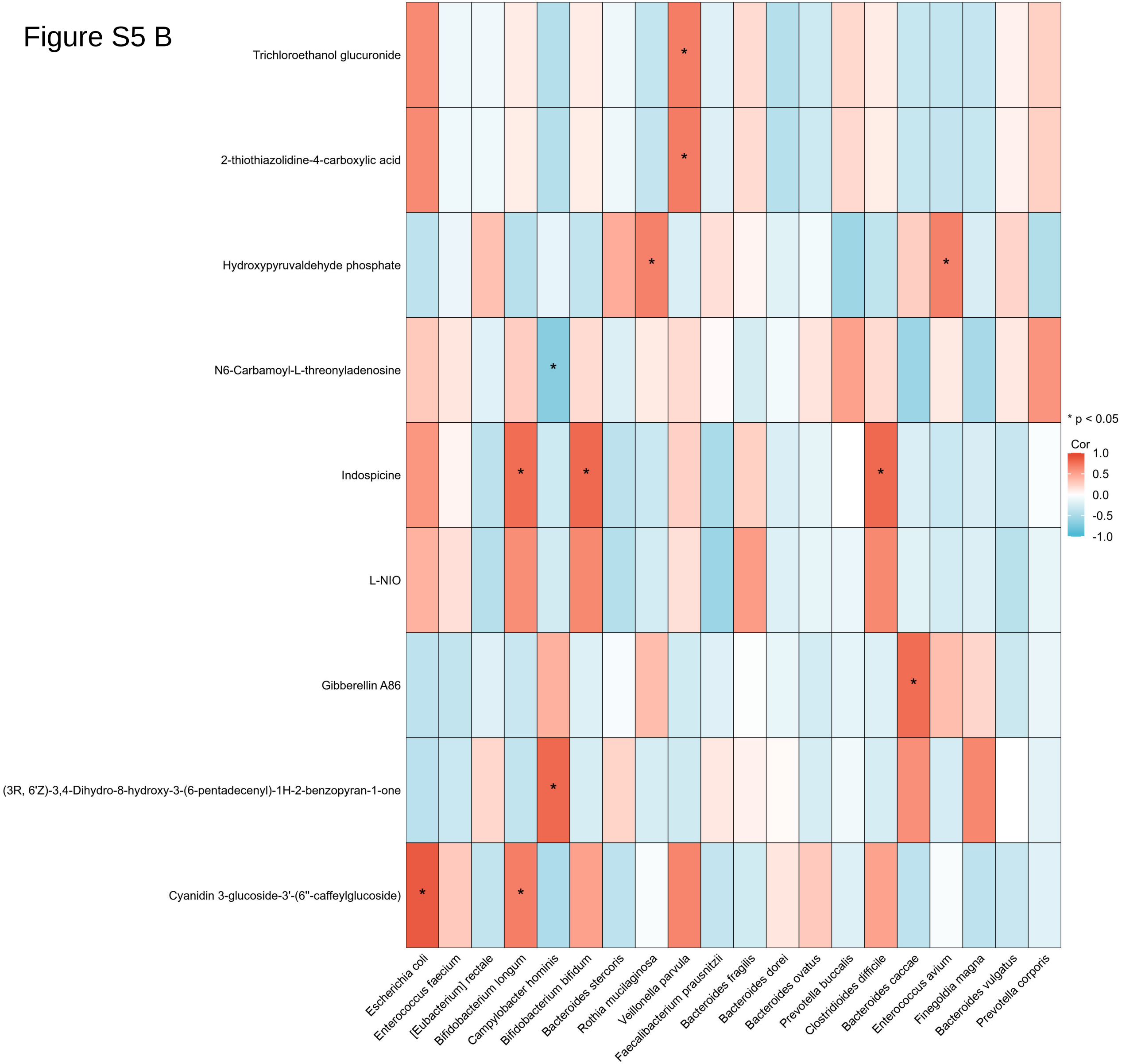
Trichloroethanol glucuronide
2-thiothiazolidine-4-carboxylic acid
Hydroxypyruvaldehyde phosphate
N6-Carbamoyl-L-threonyladenosine
Indospicine
L-NIO
Gibberellin A86
(3R, 6'Z)-3,4-Dihydro-8-hydroxy-3-(6-pentadecenyl)-1H-2-benzopyran-1-one
Cyanidin 3-glucoside-3'-(6''-caffeylglucoside)
Escherichia coli
Enterococcus faecium
[Eubacterium] rectale
Bifidobacterium longum
Campylobacter hominis
Bifidobacterium bifidum
Bacteroides stercoris
Rothia mucilaginosa
Veillonella parvula
Faecalibacterium prausnitzii
Bacteroides fragilis
Bacteroides dorei
Bacteroides ovatus
Prevotella buccalis
Clostridioides difficile
Bacteroides caccae
Enterococcus avium
Finegoldia magna
Bacteroides vulgatus
Prevotella corporis
* p < 0.05
Cor
1.0
0.5
0.0
-0.5
-1.0

Figure S5 C

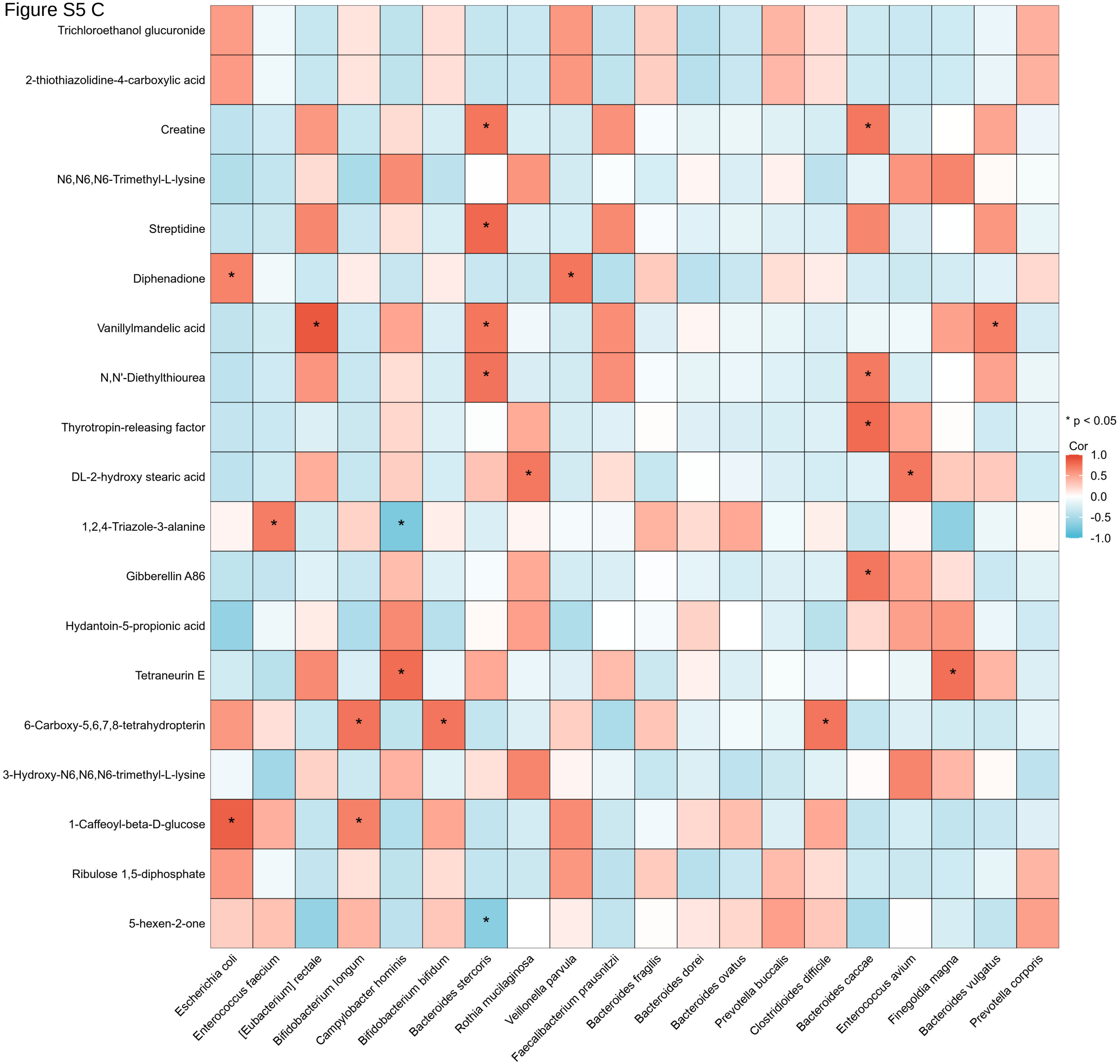
Trichloroethanol glucuronide
2-thiothiazolidine-4-carboxylic acid
Creatine
N6,N6,N6-Trimethyl-L-lysine
Streptidine
Diphenadione
Vanillylmandelic acid
N,N'-Diethylthiourea
Thyrotropin-releasing factor
DL-2-hydroxy stearic acid
1,2,4-Triazole-3-alanine
Gibberellin A86
Hydantoin-5-propionic acid
Tetraneurin E
6-Carboxy-5,6,7,8-tetrahydropterin
3-Hydroxy-N6,N6,N6-trimethyl-L-lysine
1-Caffeoyl-beta-D-glucose
Ribulose 1,5-diphosphate
5-hexen-2-one
Escherichia coli
Enterococcus faecium
[Eubacterium] rectale
Bifidobacterium longum
Campylobacter hominis
Bifidobacterium bifidum
Bacteroides stercoris
Rothia mucilaginosa
Veillonella parvula
Faecalibacterium prausnitzii
Bacteroides fragilis
Bacteroides dorei
Bacteroides ovatus
Prevotella buccalis
Clostridioides difficile
Bacteroides caccae
Enterococcus avium
Finegoldia magna
Bacteroides vulgatus
Prevotella corporis
* p < 0.05
Cor
1.0
0.5
0.0
-0.5
-1.0

Figure S5 (A) shows a Pearson’s correlation heatmap depicting the relationship between the gut microbiota at D0 and urinary metabolites at D0 (A), D1 (B), and D2 (C). A positive coefficient for the interaction term (highlighted in red) indicates higher expression levels of the gut microbiota in association with the specified factor. Conversely, a negative coefficient (displayed in blue) denotes a decrease in the gut microbiota's expression levels when the factor is present.
