# Supplementary material for: Investigating gut microbiota–blood and urine metabolite correlations in early sepsis-induced acute kidney injury: insights from targeted KEGG analyses
Source: Front Cell Infect Microbiol. 2024 Jun 3;14:1375874. doi: 10.3389/fcimb.2024.1375874 (PMC11180806; doi:10.3389/fcimb.2024.1375874)
Supplement: Supplementary file 6 [file DataSheet_6.pdf]

Figure. S6

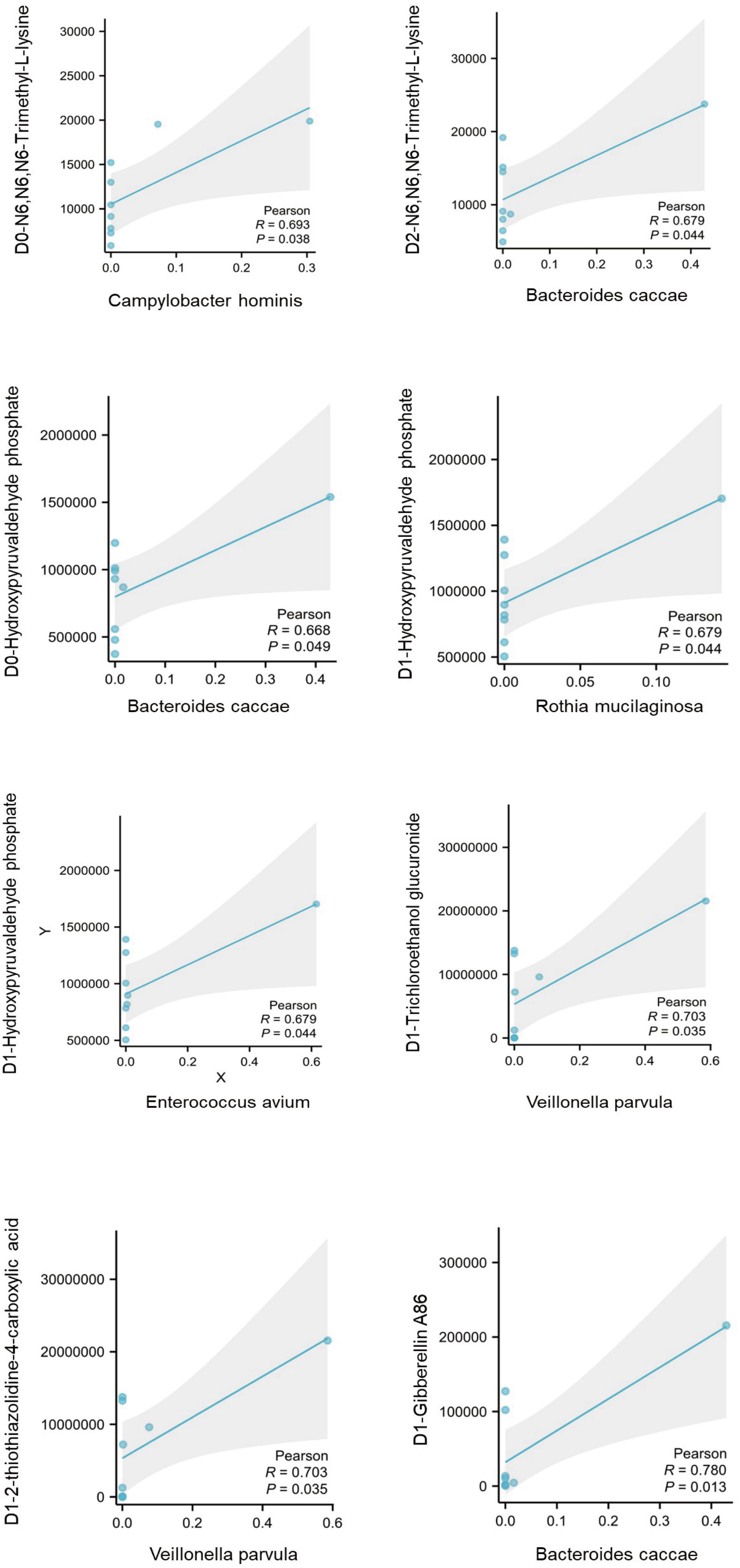

Figure S6. The fitting curves of the significant correlations between gut microbiota and blood metabolites, as well as urine metabolites.
